# Theranostic Angiopep-2-Conjugated FeTaO_*x*_@Au Core–Shell Magnetic Nanoparticles for Glioma Treatment and Dual Medical Imaging

**DOI:** 10.1021/acsabm.5c01925

**Published:** 2026-01-06

**Authors:** Kayalvizhi Samuvel Muthiah, Senthilkumar Thirumurugan, Susaritha Ramanathan, Ming-Hsuan Yeh, Udesh Dhawan, Yu-Chien Lin, Ching-Po Lin, Wai-Ching Liu, Yuan-Yun Tseng, Ching-Li Tseng, Ren-Jei Chung

**Affiliations:** † Department of Chemical Engineering and Biotechnology, 34877 National Taipei University of Technology (Taipei Tech), Taipei 10608, Taiwan; ‡ Centre for the Cellular Microenvironment, Division of Biomedical Engineering, James Watt School of Engineering, Mazumdar-Shaw Advanced Research Centre, 3526 University of Glasgow, Glasgow G116EW, U.K.; § School of Materials Science and Engineering, 54761 Nanyang Technological University, 50 Nanyang Avenue, Singapore 639798, Singapore; ∥ Institute of Neuroscience, 34914 National Yang Ming Chiao Tung University, Taipei 11221, Taiwan; ⊥ Department of Food and Health Sciences, 435815 Technological and Higher Education Institute of Hong Kong, Hong Kong 999077, Hong Kong; # Department of Neurosurgery, New Taipei Municipal TuCheng Hospital (Built and Operated by Chang Gung Medical Foundation), New Taipei City 236017, Taiwan; ∇ College of Medicine, Chang Gung University, Taoyuan 33302, Taiwan; ○ Graduate Institute of Biomedical Materials and Tissue Engineering, College of Biomedical Engineering, Taipei Medical University, Taipei City 110, Taiwan; ◆ International Ph. D. Program in Biomedical Engineering, College of Biomedical Engineering, 38032 Taipei Medical University, Taipei City 110, Taiwan; ¶ Research Center of Biomedical Device, College of Biomedical Engineering, Taipei Medical University, Taipei City 110, Taiwan; †† International Ph. D. Program in Cell Therapy and Regenerative Medicine, College of Medicine, Taipei Medical University, Taipei City 110, Taiwan; ‡‡ High-value Biomaterials Research and Commercialization Center, National Taipei University of Technology (Taipei Tech), Taipei 10608, Taiwan

**Keywords:** FeTaO_*x*_@Au-ANG, magnetic hyperthermia, glioma, MRI, computed tomography

## Abstract

In this work, iron–tantalum oxide nanoparticles (FeTaO_*x*_ NPs) with a gold coating modified by Angiopep-2 (FeTaO_*x*_@Au-ANG) were developed to achieve dual-modality imaging and magnetically induced hyperthermia therapy for glioma. The 13.5 nm sized FeTaO_*x*_@Au-ANG NPs’ exhibited superparamagnetic behavior with excellent dual *T*_1_/*T*_2_-weighted MRI contrast of Fe existence and enhanced X-ray attenuation for computed tomography (CT) imaging, enabling accurate tumor localization through complementary imaging modalities. The incorporation of Ta and Au not only improved biocompatibility but also provided a high CT contrast effect. Upon magnetic stimulation, the NPs efficiently elevated the intratumoral temperature, leading to a significant (∼90%) reduction in glioma cell viability. ANG modification further enhanced the targeted uptake of NPs by glioma cells. Immunohistochemical analysis revealed extensive coagulative and glial necrosis, elevated GFAP expression, and a reduced Ki67 index, consistent with effective tumor ablation. *In vivo*, FeTaO_*x*_@Au-ANG NPs treatment markedly suppressed tumor growth and extended survival by 18 days. Overall, this multifunctional nanoplatform demonstrates synergistic MRI/CT imaging-guided magnetic hyperthermia with high therapeutic efficacy and minimal side effects, offering strong potential for clinical cancer theranostics.

## Graphical abstract

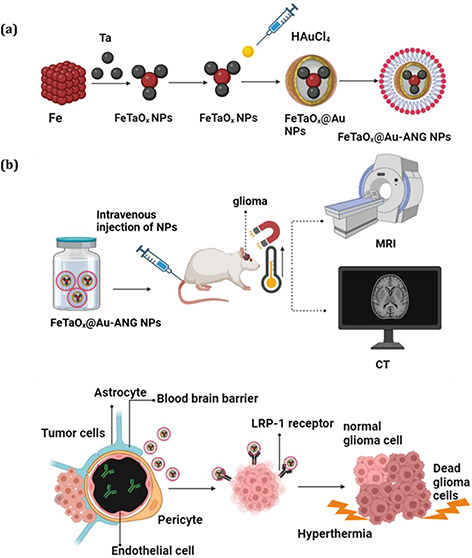

## 1 Introduction

Gliomas account for 81% of malignant brain tumors and are the most invasive intracranial neoplasms of the central nervous system. Glioma is also the second largest cause of cancer-associated death among teenagers.^1–3^ Glioma is distinguished by significant morbidity, dismal prognosis, mortality rates, and a short survival duration of approximately 15 months.^4^ Chemotherapy is a clinically accepted treatment strategy for glioma.^5^ This is because typical surgical resection does not entirely prevent the invasion of gliomas into the surrounding tissue.^2,6^ However, the effectiveness of glioblastoma chemotherapy is hindered because of its low permeability across the blood-brain barrier (BBB)^7–9^ and its low availability at the tumor site.^10–14^ Although photothermal therapy (PTT) is considered the paragon of precision localized therapy, widely applied in clinics, it is still hindered by the major drawback of limited tissue penetration (∼1 cm) under laser irradiation. As another hyperthermia modality, magnetic hyperthermia (MHT) is more widely applicable than PTT and is capable of eliminating deep-seated tumors under an alternating magnetic field (AMF) without any limits on tissue penetration.^15^ Moreover, MHT is considered the best therapeutic tool for internal tumors (e.g., brain, liver, pancreas) due to uniform heating, a less invasive setup, reduced phototoxicity, and better compatibility with imaging compared to PTT. Both magnetism and light can induce responsive materials to generate heat; magnetism can achieve greater penetration depth than light, and does not produce toxicity or deactivation phenomena like light.^16,17^ In summary, MHT overcomes the major drawbacks of chemotherapy and PTT by enabling deep, uniform, and controllable heating with minimal side effects. Its multifunctional capability for imaging and combined therapies makes it a promising strategy for precise and effective tumor treatment.

Low-density lipoprotein receptor-related protein (LRP) is highly expressed in both the BBB and gliomas.^18,19^ LRP is considered a possible therapeutic target for gliomas. Angiopep-2 (ANG; TFFYGGSRGKRNNFKTEEY) is a 2.4 kDa protein that is a specific ligand for LRP. ANG readily transits the BBB into the brain, where ANG facilitates nanoparticles (NPs) transport across the BBB via LRP-mediated receptor transcytosis. After binding to LRP on brain endothelial cells, the ANG–NPs complex undergoes endocytosis, intracellular trafficking, and exocytosis into the brain parenchyma. Since LRP is also overexpressed on glioma cells, ANG further enhances tumor-specific uptake, improving targeted drug delivery and therapeutic efficacy. Therefore, ANG has been extensively used to modify the surface of NPs so that the engineered NPs can cross the BBB and reach gliomas to improve treatment efficacy.^20–25^

Magnetic NPs (MNPs) are inorganic and zero-dimensional materials with metal-based configurations. These NPs can be easily manipulated using an AMF and are utilized in various applications. MNPs also display intrinsic and unique features, such as high saturation magnetization (Ms), biocompatibility, and low toxicity. The research interest reflecting these beneficial attributes has led to advancements that include industrial, environmental, analytical, and biomedical applications.^26–28^ In general, MNPs possess both *in vitro* and *in vivo* biomedical applications. For instance, *in vitro* applications are predominantly utilized in diagnostic procedures, including separation/selection, magnetic relaxometry, and magnetic resonance imaging (MRI),^29^ whereas *in vivo* applications include diagnostic techniques, such as nuclear MRI, and therapeutic procedures that include medication delivery and MHT.^30^ Generally, Iron oxide (Fe_3_O_4_ and Fe_2_O_3_) NPs have been extensively explored by researchers owing to their ability to become nonmagnetic when the magnetic field is removed. They can be easily functionalized with polymers and other materials, and are widely used for *in vitro* diagnostics. Magnetic field parameters, including amplitude, frequency, and duration, play an essential role in MNPs-mediated cytotoxicity.^31^

In this study, we developed a safe and self-targeted nano therapeutic approach to treat glioma. MNPs with high Ms values have been utilized to generate heat for MHT.^32^ Technically, MNPs can be administered either locally or intravascularly and are controlled by an external AMF. This approach, known as MHT, generates heat inside the target cells by focusing on magnetic fields.^33^ Busch^34^ and Coley et al.^35^ described the disappearance of sarcomas in individuals experiencing extremely high fever associated with the response of the immune system to bacterial infection. Other studies demonstrated that cancer cells are vulnerable to high temperatures, with their proliferation significantly inhibited at temperatures ranging from 41 to 47 °C for a minimum of 20–60 min.^36,37^ While this response has therapeutic potential and has been substantially refined, ongoing challenges include the rapid development of adverse side effects in healthy cells, such as blisters, burns, and pain throughout the body. In contrast, hyperthermia is applied locally instead of exposing the entire body to high temperatures. This reduces unwanted side effects and improves therapeutic efficacy.^38^ Aggregation of particles due to the attractive interparticle forces in colloidal fluids is another problem that hinders therapeutic applications. These interparticle forces include dipole–dipole interactions, van der Waals forces, Brownian effects, and electrostatic forces.^39^ The aggregation of NPs results in a slower rate of magnetization fluctuations, rendering the material inappropriate for use in MHT. Studies report Au shows surface plasmon resonance under UV/NIR light; this work focuses on MHT driven by the Fe core’s superparamagnetism, as Au is nonmagnetic. The thin, uniform Au coating preserves magnetic heating efficiency, and ANG enables targeted delivery across the BBB.^40–42^ MHT-based applications that are effective for glioma treatment require stable solutions of MNPs in a physiological medium, such as water or phosphate-buffered saline (PBS).

Moreover, in glioma therapeutics, the drug delivery process in brain tumors is a double-edged sword. To overcome these limitations, novel core–shell-structured iron tantalum oxide (FeTaO_*x*_) NPs conjugated with gold (Au) and angiopep-2 (ANG) were developed (FeTaO_*x*_@Au-ANG NPs) to treat gliomas. The presence of ANG helps to target the tumor sites where the cell-penetrating peptide (CPP) triggers NPs to cross the BBB. Furthermore, the use of Fe-based MNPs enables the generation of a localized hyperthermic effect, which leads to tumor cell apoptosis. The inclusion of Fe in NPs makes them highly effective as dual MRI (*T*_1_/*T*_2_) contrast agents. The incorporation of Au enhances the capability of the NPs as contrast agents in computed tomography (CT), allowing dual imaging. The internalization and effectiveness of FeTaO_*x*_@Au-ANG NPs’ MHT potency have been demonstrated to inhibit glioma growth in both *in vitro* and *in vivo* (Scheme 1). The synergistic application of the synthesized FeTaO_*x*_@Au-ANG NPs specifically targets glioma cells and induces programmed cell death. Images of the progression of tumor therapy are valuable for nanotheranostic use.

**Scheme 1:**
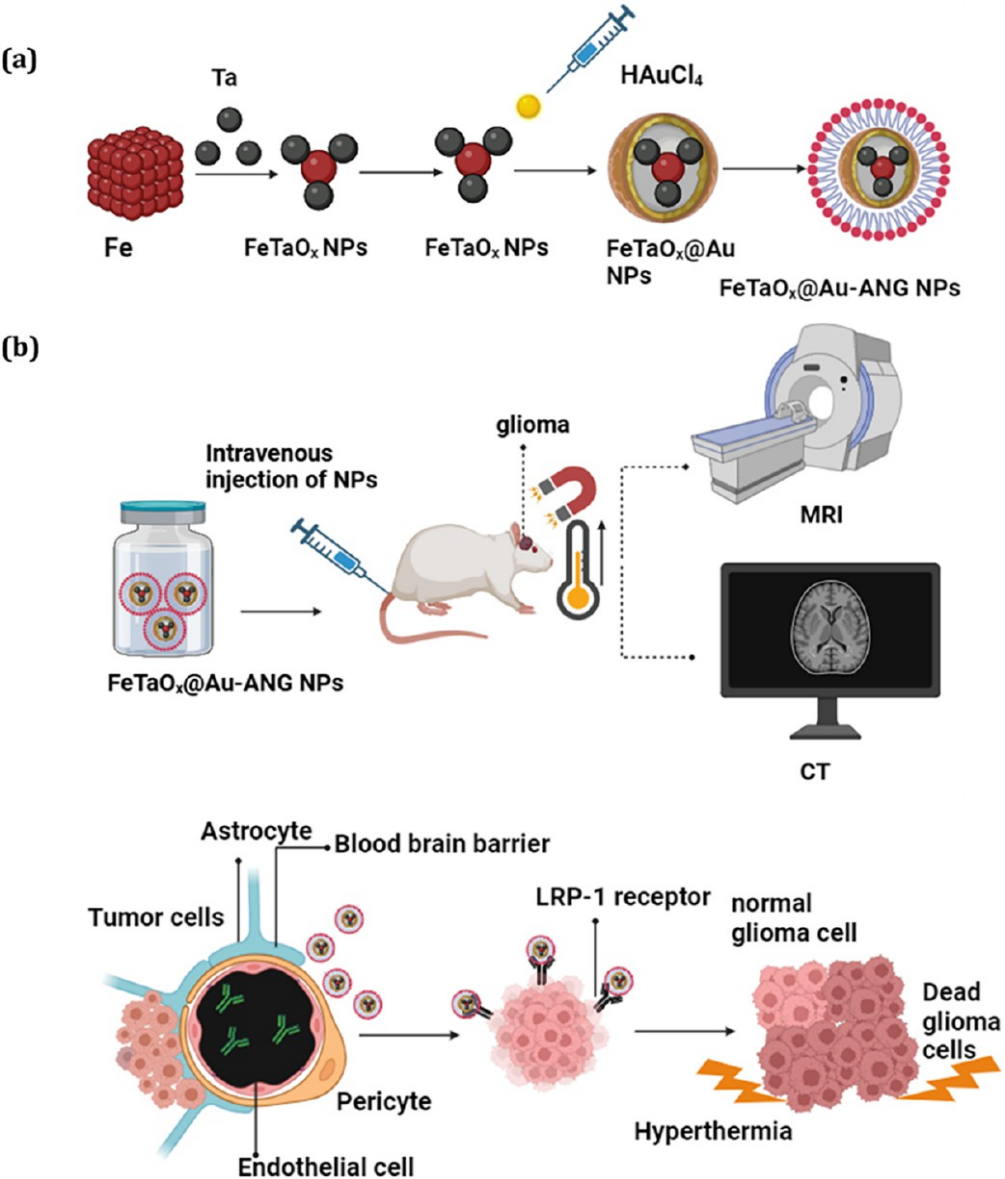
Schematic Diagrammatic Representation of the Magnetic-Hyperthermia Application of the FeTaO_*x*_@Au NPs Modified with Angiopep-2 (ANG) for Magnetothermal Therapy of Brain Tumor is Given Here^a^

a (a) Represents the synthesis procedure of the NPs, (b) shows the dual-modal imaging property and the mechanism of glioma apoptosis of the synthesized NPs.

The present findings reinforce the potential value of FeTaO_*x*_@Au-ANG NPs in various cancer research, such as drug delivery, drug discovery, and theranostics.

## 2 Materials and Methods

### 2.1 Materials

Tantalum tetrachloride (TaCl_4_), sodium borohydride (NaBH_4_), ferrous sulfate (FeSO_4_), polyvinylpyrrolidone (PVP), chloroauric acid (HAuCl_4_), polyethylenimine (PEI), PBS, and 99.9% pure ethanol (C_6_H_5_OH) were acquired from Sigma-Aldrich USA or Merck, Germany. ANG was provided by Qunda Marine Technology Co., Ltd.

### 2.2 Characterization

Morphological analysis and the size distribution of FeTaO_*x*_, FeTaO_*x*_@Au, and FeTaO_*x*_@Au-ANG NPs, providing a more intuitive and statistically meaningful measurement of particle size, were performed using transmission electron microscopy (TEM). A total of 100 nanoparticles were measured for each sample. Zeta potential curves were obtained to determine the specific area and pore size of the NPs conjugated with the peptide. The crystalline structure of the synthesized FeTaO_*x*_@Au-ANG NPs was analyzed using X-ray diffraction (XRD). Compositions of the prepared NPs were examined using energy-dispersive X-ray spectroscopy (EDS) and Inductively Coupled Plasma Optical Emission Spectroscopy (ICP-OES). The decoration of ANG with FeTaO_*x*_@Au NPs was analyzed using Raman spectroscopy, zeta potential, and Fourier transform infrared spectroscopy (FTIR). Various orbital ranges in the prepared NPs were predicted using X-ray photoemission spectroscopy (XPS). The magnetic behavior of FeTaO_*x*_@Au-ANG NPs was investigated using a superconducting quantum interference device (SQUID).

### 2.3 Preparation of FeTa NPs

Iron–tantalum (FeTa) NPs were prepared using a facile hydrothermal method. Initially, 0.5 g of PVP solution was added to 20 mL (99.5%) of ethanol in a beaker and vigorously stirred for 15 min. Aqueous solutions of TaCl_4_, FeSO_4_, and NaBH_4_ (0.02 M) were also prepared. Afterward, 10 mL of ethanol was added to 0.15 g of TaCl_4_, 20 mL of distilled water (DI H_2_O) to 0.3 g of NaBH_4,_ and 0.117 g of FeSO_4_. The prepared metal aqueous solutions were mixed with the PVP solution and ultrasonicated until complete dispersion was achieved. The dispersed solution was transferred to a Teflon-lined flask and sealed tightly. The airtight flask was placed in an oven and heated to a temperature of 180 °C within 30 min. This temperature was maintained for approximately 18 h. The samples were then cooled to 35 °C within 30 min.

### 2.4 Purification of FeTaO_*x*_ NPs

After completion of the hydrothermal process, the solution was allowed to reach a room temperature of 25 °C. The Teflon flask was removed from the oven, and the solution was centrifuged at 9000 rpm for 10 min. The obtained MNPs were positioned in a magnetic field established using a 4000 Gaussian magnet, kept in an ultrasonic vibrator tank with 20 mL of anhydrous alcohol and 10 mL of secondary water, and centrifuged at 9000 rpm for 10 min. The collected NPs were uniformly dispersed in the solvent and centrifuged three times at 9000 rpm to wash the NPs. The final supernatant was removed, and the NPs that had collected on the wall of the tube were dried for 3 h at room temperature using a vacuum pump. A black powder was obtained, representing iron–tantalum oxide (FeTaO_*x*_) NPs.

### 2.5 Au Surface Modification of FeTaO_*x*_ NPs

The FeTaO_*x*_ NPs (10 mg) were transferred to a 50 mL centrifuge tube, dissolved in 10 mL of secondary water, and ultrasonically dispersed for 15 min. 3 mL of HAuCl_4_, 0.3 mL of PEI, and 4 mL of NaBH_4_ were added and dispersed by sonication for 10 min. The mixture was then centrifuged at 9000 rpm for 10 min and washed three times by centrifugation under the same conditions to purify the NPs. The NPs were vacuum-dried for 3 h at room temperature to obtain black FeTaO_*x*_@Au NPs.

### 2.6 ANG Surface Modification of FeTaO_*x*_@Au NPs

To surface-modify FeTaO_*x*_@Au NPs, 5 mg of ANG and 10 mg of FeTaO_*x*_@Au NPs were recovered following centrifugation in a 50 mL tube and dispersed in 20 mL PBS. The mixture was transferred to a three-necked bottle, covered with a balloon under an inert atmosphere, and stirred for 24 h. The obtained NPs were washed with PBS, sonicated, and centrifuged (9000 rpm for 10 min) to yield FeTaO_*x*_@Au-ANG NPs.

### 2.7 Concentration-Dependent Temperature Elevation of FeTaO_*x*_@Au-ANG NPs

To investigate the potency of the prepared FeTaO_*x*_@Au-ANG NPs in generating heat upon magnetic stimulation, the NPs were dispersed at different concentrations (0.625, 1.25, 2.5, 5, and 10 mg/mL) in DI water and placed in a 1.5 mL microcentrifuge tube. The samples were exposed to an AMF of 700–1100 kHz (Power cube 3.2 kw, HF2; President Honor Industries Co., Ltd.) for 10 min. The temperatures of the samples were recorded every 30 s.

### 2.8 Stability of NPs

FeTaO_*x*_@Au-ANG NPs were dissolved in PBS solution with a concentration of 1 mg/mL, and incubated for different time intervals from 0 to 24 h. After the incubation process, the respective TEM images were taken to check the morphology changes.

### 2.9 *In Vitro* CT and MRI Imaging

The efficacy of the designed FeTaO_*x*_@Au-ANG NPs for dual-modality CT and MRI imaging in medical applications was explored using a Skyscan 1076 device for CT and a 7T positron emission tomography/MRI device (Bruker). For CT and MRI, FeTaO_*x*_@Au-ANG NPs were prepared at different concentrations (0.03125, 0.0625, 0.125, 0.25, and 0.5 mg/mL) and dissolved in 0.5% agar in 1.5 mL microcentrifuge tubes. The solutions were subjected to 7T magnetic resonance spectroscopy using a repetition time of 5000 ms, and time to echo of 60 ms, and microCT. For MRI, modulation of both relaxation times (*T*_1_ and *T*_2_) was obtained because of concentration-dependent contrast variation. The CT data revealed that the image became brighter when the concentration of NPs increased, suggesting that the effect was concentration-dependent.

### 2.10 Cell Culture

C6 rat glioma cells (CCL107; ATCC) and L929 mouse fibroblasts (CCL1; ATCC) were grown in Dulbecco’s modified Eagle’s medium (DMEM) supplemented with 10% fetal bovine serum (GIBCO). Cells were cultured at 37 °C in an atmosphere of 5% CO_2_ and 95% humidity.

### 2.11 *In Vitro* Cytotoxicity Analysis

The *in vitro* cytotoxicity was determined using a cell counting kit (CCK-8). C6 and L929 cells were seeded at a density of 1 × 10^5^ cells/mL in 96-well plates and incubated for 24 h in a CO_2_ atmosphere. After 24 h, C6 and L929 cells were treated with various concentrations (1000, 500, 250, 125, 62.5, and 31.25 μg/mL) of synthesized FeTaO_*x*_@Au-ANG NPs and incubated for 24 h. The next day, each well received 10 μL of the CCK-8 reagent, followed by incubation for 1–2 h. Absorbance at 450 nm was measured using a microplate reader. The cytotoxicity was assessed using a standard CCK-8 assay.

### 2.12 Magnetic Hyperthermia Effect on C6 Cells

C6 cells were seeded at a density of 3 × 10^6^ cells/mL in a 35 mm dish and incubated for 24 h. The cells were then treated with FeTaO_*x*_, FeTaO_*x*_@Au, and FeTaO_*x*_@Au-ANG NPs at different concentrations. After 6 h of incubation, the cells were exposed to AMF for 5 min and then treated with 10 μL of the CCK-8 reagent. The percentage of cell viability was determined.

### 2.13 Endocytosis of Prepared FeTaO_*x*_@Au-ANG NPs

The intracellular uptake of NPs by glioma cells was detected using optical microscopy. C6 cells were seeded at a density of 3 × 10^6^ cells/mL in a 35 mm dish and incubated for 24 h. The cells were then treated with FeTaO_*x*_, FeTaO_*x*_@Au, and FeTaO_*x*_@Au-ANG NPs (250 μg/mL) for 1–4 h. Intracellular uptake of NPs was observed using an optical microscope.

### 2.14 Cellular Uptake of FeTaO_*x*_@Au-ANG NPs by ICP-OES

To investigate the NPs’ uptake in C6 cancer cells and L929 normal fibroblasts, the cells were seeded in 35 mm diameter Petri dishes at a density of 3 × 10^6^ cells/mL and incubated for 24 h. The NPs (FeTaO_*x*_, FeTaO_*x*_@Au, and FeTaO_*x*_@Au-ANG) were sterilized by ultraviolet radiation for 30 min, purified, and diluted in DMEM to a final concentration of 250 μg/mL. After 24 h of incubation, 1 mL of dilute FeTaO_*x*_, FeTaO_*x*_@Au, and FeTaO_*x*_@Au-ANG NPs was added to a culture dish containing normal or glioma cells for 0.5 or 2 h. The samples were washed with PBS and detached using trypsin and ethylenediaminetetraacetic acid, digested using concentrated nitric acid, and the Fe concentration was determined by ICP-OES.

### 2.15 Analysis of *In Vivo* MRI Properties of Prepared NPs

Sprague–Dawley (SD) rats with preformed C6 cell brain tumors were purchased from BioLASCO (Taiwan). The rats were divided into a control group and groups treated with FeTaO_*x*_, FeTaO_*x*_@Au, and FeTaO_*x*_@Au-ANG NPs. MRI images of the tumor-bearing rats were taken before the administration of the NPs. Rats were injected via the tail vein with 200 μL PBS (control) and 200 μL of FeTaO_*x*_@Au-ANG NPs (2.5 mg/mL) prepared in 0.01 M of PBS. All rats were analyzed using an Nb–Fe–B magnet (0.5 T) with a diameter of 2 cm × 2 cm that was fixed to the skull. Two hours after injection, an MRI was performed to compare the signal intensities.

### 2.16 *In Vivo* Animal Model Studies

SD rats were used for *in vivo* studies. Before starting the experiment, the rats were acclimated for 1 week, during which they were provided with free access to food and water. All animal experiments were conducted using protocols approved by the relevant committee of Taipei Medical University (approval LAC-2017–0485). Typically, 15 SD rats were used in the experimental procedure. For anesthesia, a solution of Zoletil 50 and Rompun (1:2) was prepared and 0.40 cc was administered abdominally. Subsequently, a specific region of the rat head was shaved and sterilized using alcohol. A 2 cm dissection was made with a scalpel 1.5 cm from the eye. A hole (1 cm × 1 cm) was drilled in each skull with a high-speed drill bit. The skull of each rat was fixed in a stereotactic frame, and 5 μL of a C6 cell suspension (5 × 10^5^ cells) was injected into the soft tissue.

### 2.17 Evaluation of Tumor Growth

To assess the use of FeTaO_*x*_@Au-ANG NPs for cancer theranostics, SD rats were allocated into four groups: control, FeTaO_*x*_ NPs (2.5 mg/mL) + AMF, FeTaO_*x*_@Au NPs (2.5 mg/mL) + AMF, and FeTaO_*x*_@Au-ANG NPs (2.5 mg/mL) + AMF. In the control group, 0.01 M of PBS was administered via tail injection. For the other experimental groups, 200 μL of the NPs (2.5 mg/mL) was intravenously administered to the rats. An Nb–Fe–B magnet (0.5 T) with a diameter of 2 cm × 2 cm was placed on the skull of each rat. To generate magnetic stimulation 2 h after administration, the rat’s head was covered with a high-frequency heating coil (700–1100 kHz) for 15 min. Magnetic therapy was administered once a week, and tumor volumes were measured on days 0, 7, and 14. After day 14, brain tumor sections were examined to validate the effect of magnetic therapy on the tumor areas.

### 2.18 Inductively Coupled Plasma Optical Emission Spectroscopy (ICP-OES) of Major Organs

For ICP-OES analysis, the major organs (heart, liver, spleen, lung, brain, and kidney) were digested using 9 mL 65% HNO_3_ and 1 mL 30% HCl for 20 min at 180 °C using a microwave digestion apparatus. To reduce the HNO_3_ concentration, the above solution was diluted 10 times with DI H_2_O after the digestion process. The concentration of the elements (iron (Fe), and tantalum (Ta)) in each organ was detected by ICP-OES.

### 2.19 Immunohistochemical (IHC) Analysis of Tumor Tissues and Vital Organs

IHC analyses were performed to evaluate the biocompatibility of the prepared NPs. Hematoxylin and eosin (H&E) staining was performed using 8-μm slices of the tumor. Tissues (lung, spleen, liver, kidney, and heart) were preserved in 4% paraformaldehyde, dried, and H&E-stained, followed by glial fibrillary acidic protein (GFAP), Ki67, and terminal deoxynucleotidyl transferase dUTP nick end labeling (TUNEL) staining.

### 2.20 Statistical Analysis

All the data are expressed as mean ± standard deviation. Data analysis was performed by analysis of variance (Fischer test). Significant, moderately significant, and highly significant differences were indicated by *p* < 0.05 (* in figures), <0.01 (** in figures), and <0.001 (*** in figures), respectively.

## 3 Results and Discussion

### 3.1 Characterization of FeTaO_*x*_@Au NPs Conjugated with ANG

TEM images elucidate the morphological behavior of the synthesized FeTaO_*x*_@Au-ANG NPs. Figure 1(a–c) depicts the TEM images of FeTaO_*x*_, FeTaO_*x*_@Au, and FeTaO_*x*_@Au-ANG NPs, which confirms the uniform spherical shape and their respective core–shell structure with the successful ANG surface modification of the NPs shown inFigure [Fig fig1]b,c. Figure 1(d–f) also represents the HR-TEM images of the FeTaO_*x*_, FeTaO_*x*_@Au, and FeTaO_*x*_@Au-ANG NPs, confirming the synthesized NPs’ core–shell structure. The respective particle size profiles of FeTaO_*x*_, FeTaO_*x*_@Au, and FeTaO_*x*_@Au-ANG NPs are given in Figure 1g–i, which shows that the average particle size of FeTaO_*x*_, FeTaO_*x*_@Au, and FeTaO_*x*_@Au-ANG NPs falls around 10.0 ± 0.15, 12.1 ± 1.55, 13.7 ± 0.97 nm, highlighting the successful formation of ANG-coated FeTaO_*x*_@Au NPs. From EDX studies (Figure S1), the existence of the various elements along with the elemental weight percentage are enlisted here, Fe (6.2 wt %), Ta (32.2 wt %), Au (34.1 wt %), O (16.7 wt %), C (9.6 wt %), N (0.1 wt %), and S (0.1 wt %) in FeTaO_*x*_@Au-ANG NPs respectively in Figure 1(j–q).

**Figure 1:**
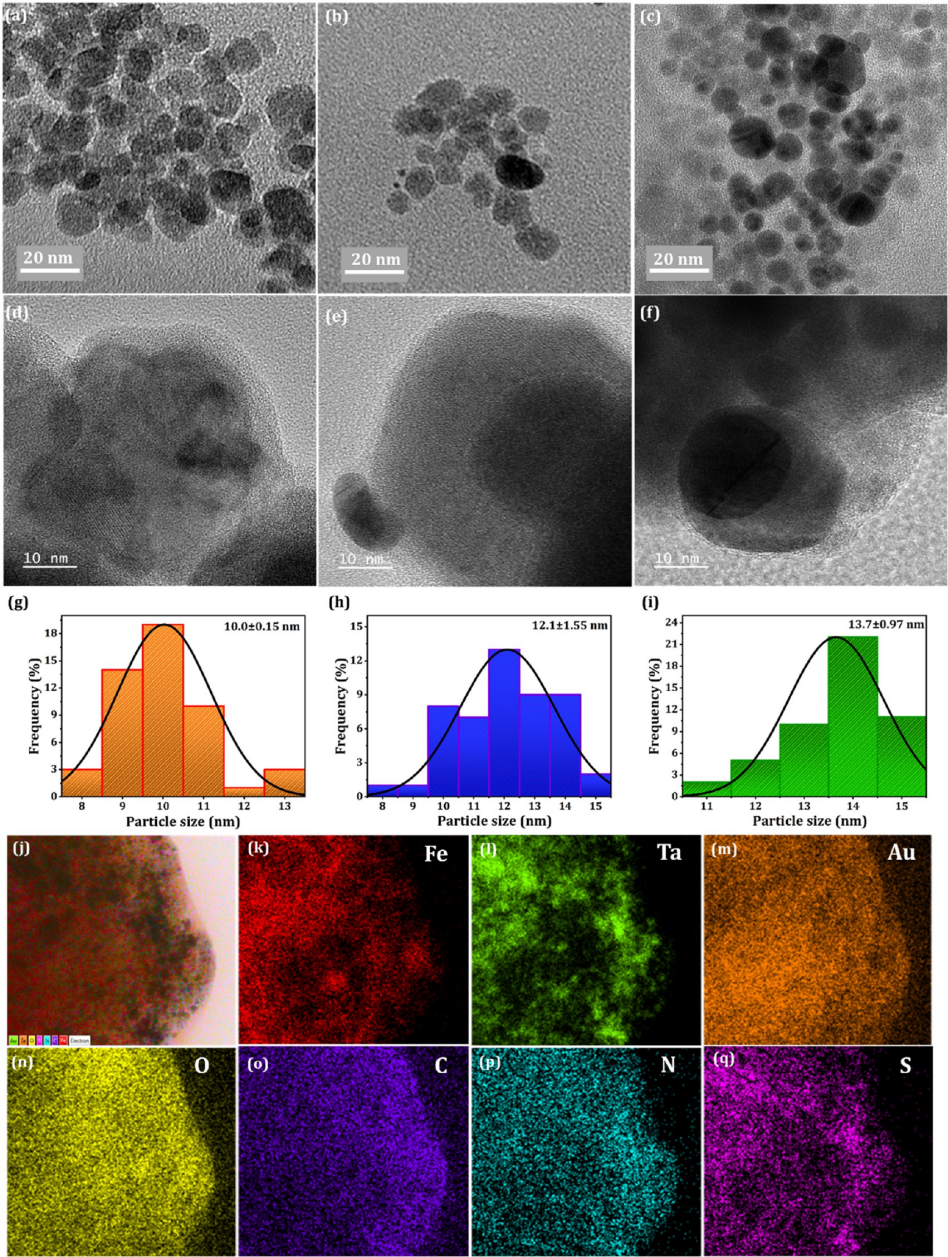
Characterization of the prepared NPs. TEM images of (a) FeTaO_*x*_ NPs, (b) FeTaO_*x*_@Au NPs, and (c) FeTaO_*x*_@Au-ANG NPs. (d) HR-TEM images of FeTaO_*x*_ NPs, (e) FeTaO_*x*_@Au NPs, and (f) FeTaO_*x*_@Au-ANG NPs. TEM–based particle size distribution analysis of (g) FeTaO_*x*_, (h) FeTaO_*x*_@Au, and (i) FeTaO_*x*_@Au-ANG NPs using Image-J software. A total of 100 nanoparticles were measured for each sample. Elemental mapping images of FeTaO_*x*_@Au-ANG NPs, (j) combined image, (k) iron (Fe), (l) tantalum (Ta), (m) gold (Au), (n) oxygen (O), (o) carbon (C), (p) nitrogen (N), and (q) sulfur (S).

Figure 2a presents the XRD graph of the FeTaO_*x*_@Au-ANG NPs. The appearance of peaks at 2θ values of 38.3°, 44.6°, and 64.8° corresponds to the planes (111), (200), and (220), highlighting the crystalline facets of Au (JCPDS 01–1174).^43^ Moreover, the crystalline facets of Fe_3_O_4_ (JCPDs 03–0863)^44^ are illustrated by the obtained peaks of 35.4°, 43.3°, 57.1°, and 51.3° corresponding to the crystal planes of (311), (400), (511), and (440) respectively. The appearance of the peak at 2θ values of 29.4°, and 36.9°, corresponding to the crystal planes of (200), and (111), validates the existence of Ta_2_O_5_ (JCPDS 18–1304).^45^ Furthermore, no peaks for pure tantalum and iron were not observed, confirming the tantalum and iron are oxidized forming Ta_2_O_5_ and Fe_3_O_4_.^44,46^ Figure 2b shows the FT-IR spectrum of FeTaO_*x*_@Au-ANG NPs, the existence of an absorption band at 1045 cm^–1^ presents the stretching vibration of C–O, the peak at 1646 cm^–1^ and 3400 cm^–1^ arises because of the occurrence of C=O, N–H, and O–H bonds in the NPs.^47^ These results highlight the successful surface modification of ANG to NPs. Furthermore, the availability of a peak at 290 cm^–1^ in the Raman spectrum (Figure 2c) elucidates the bond occurrence between gold and thiol of cystine which is not seen in FeTaO_*x*_ and FeTaO_*x*_@Au NPs, which confirms the ANG conjugation to NPs.^47^ Figure 2d represents the zeta potential analysis of the FeTaO_*x*_, FeTaO_*x*_@Au, and FeTaO_*x*_@Au-ANG NPs; the surface potential of bare ANG and FeTaO_*x*_ is calculated as 16.36 ± 0.22 mV and −30.27 ± 0.59 mV. Here, the surface potential decreases to −33.33 ± 0.25 mV after Au coating, and ultimately after coating with ANG the surface potential further decreases to −15.80 ± 0.46 mV. Negatively charged nanoparticles are considered safe for biological applications as they can evade the immune response of monocytes and macrophages in the bloodstream. The stability of the NPs is investigated by incubating the prepared NPs in the PBS solution for different time intervals (0, 6, 12, and 24 h), and the respective TEM images were taken where no changes in the morphological structure occur, which highlights the NPs’ stability (Figure S2).

**Figure 2:**
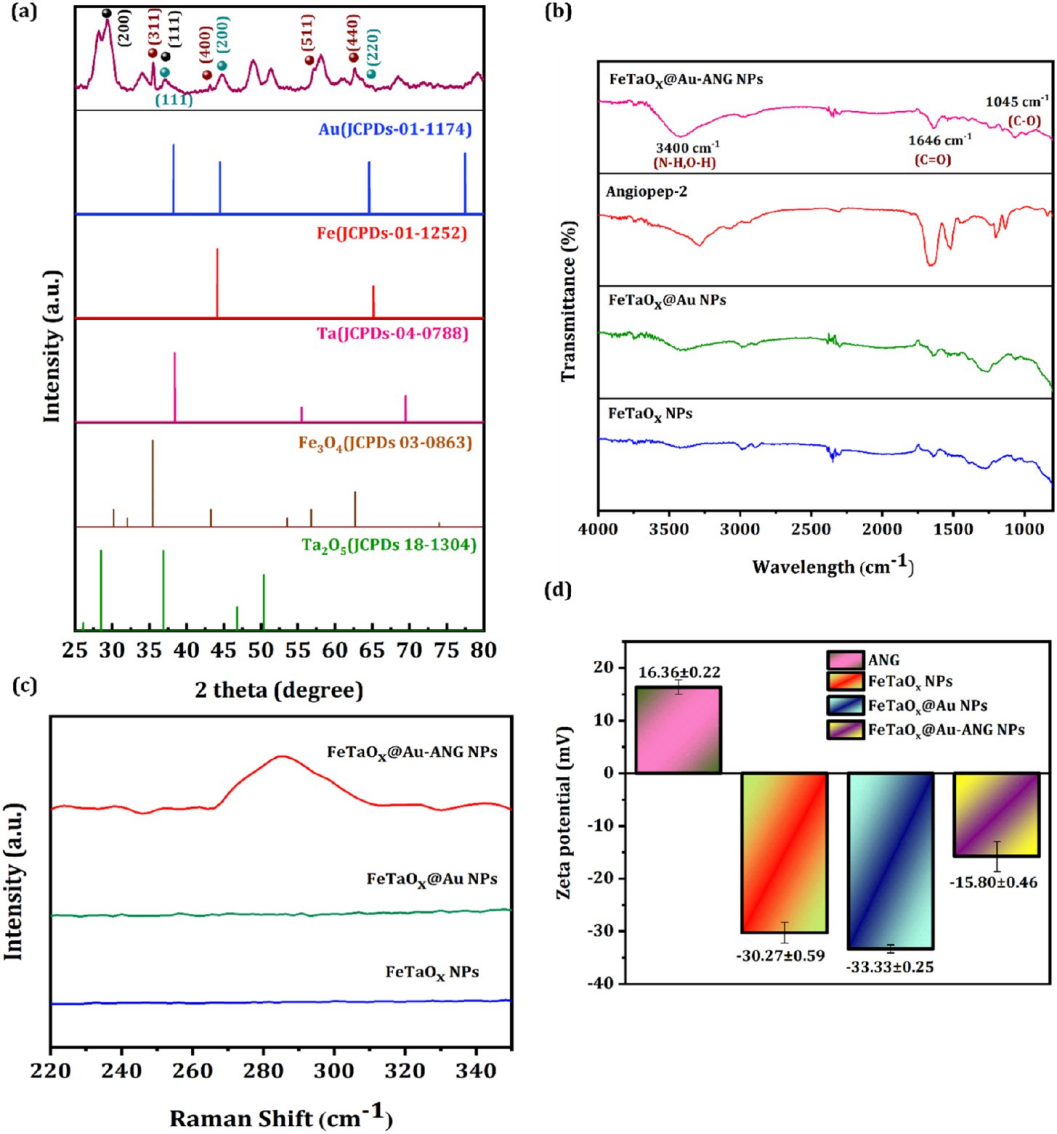
Characterization of NPs. (a) XRD analysis of FeTaO_*x*_@Au-ANG NPs. (b) FTIR spectra of ANG, FeTaO_*x*_, FeTaO_*x*_@Au, and FeTaO_*x*_@Au-ANG NPs. (c) Raman spectra before and after modification of FeTaO_*x*_, FeTaO_*x*_@Au, and FeTaO_*x*_@Au-ANG NPs. (d) Zeta potentials of ANG, FeTaO_*x*_, FeTaO_*x*_@Au, and FeTaO_*x*_@Au-ANG NPs.

XPS spectrum is used to study the elemental composition, chemical bonding, and electronic structure of FeTaO_*x*_@Au-ANG NPs. Figure 3a shows the survey spectrum of the FeTaO_*x*_@Au-ANG NPs, where the presence of core-level scans of Fe, Ta, Au, and O. The analysis of the deconvoluted Fe 2p spectra (Figure 3b) reveals three distinct peaks at 708.7, 711.5, and 723.2 eV, highlighting critical electronic structure information essential for understanding iron’s chemical state. These peaks are attributed to the existence of Fe^3+^ in the synthesized NPs. The peaks at 711.51 and 723.9 eV convey the presence of Fe^2+^ valency.^48^ The XPS plot of tantalum shows the presence of two peaks at 23.5 and 25.4 eV, highlighting the occurrence of Ta 4f_5/2_ and Ta 4f_7/2_ corresponding to Ta^3+^ (Figure 3c).^49^ Figure 3d depicts the deconvoluted spectrum of Au 4f; the existence of binding energies of 87.4 and 83.7 eV corresponds to the Au^0^ and Au^3+^ states.^50^ The O 1s binding energy peak in Figure 3e shows peaks at 527.6 and 529 eV, indicating the presence of oxygen and confirming the successful conjugation of ANG to FeTaO_*x*_@Au NPs.^51^

**Figure 3:**
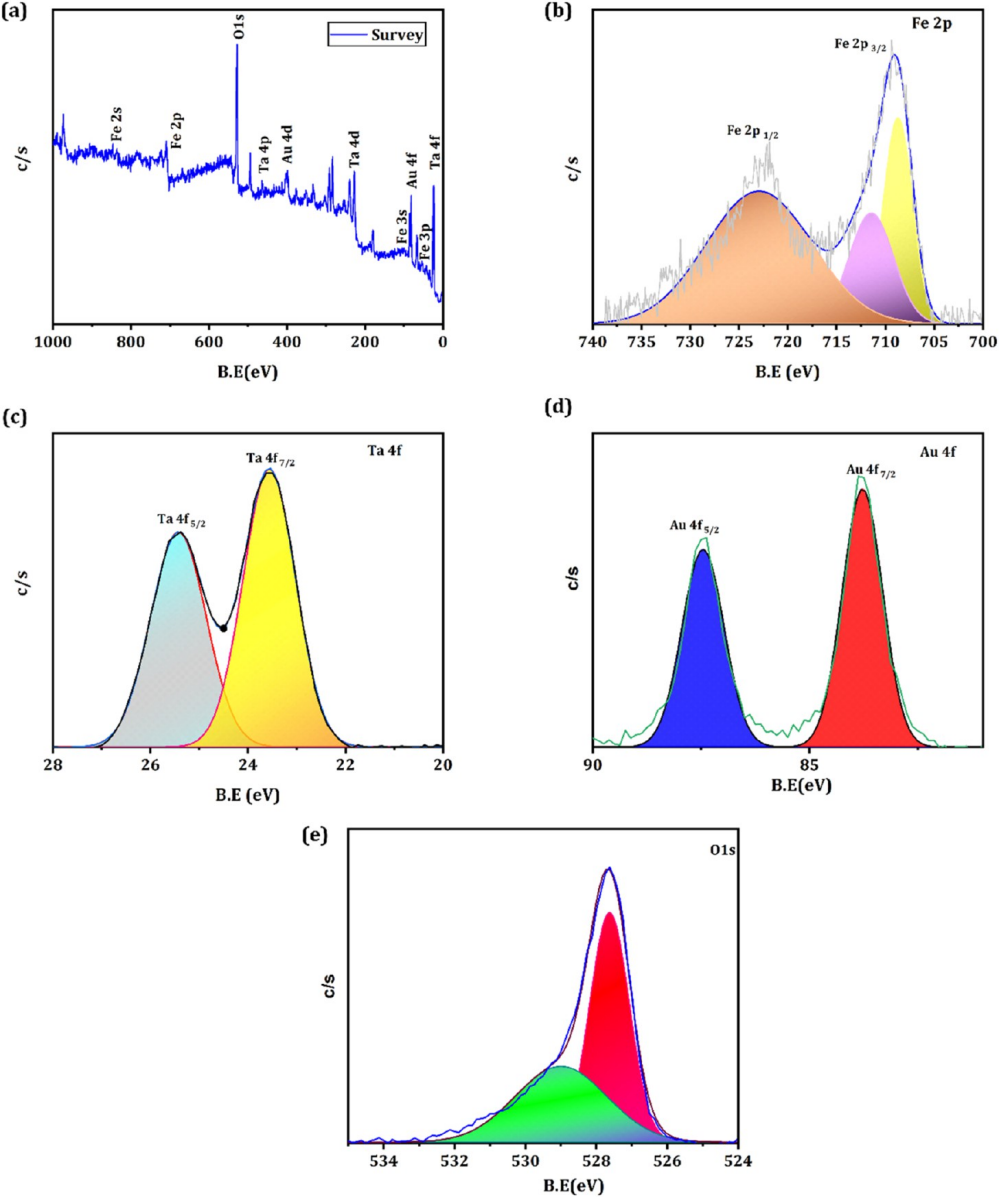
(a) XPS survey spectrum of FeTaO_*x*_@Au-ANG NPs. (b–e) represents the core level spectrum of Fe, Ta, Au, and O, respectively.

Figure 4a presents the SQUID analysis of FeTaO_*x*_, FeTaO_*x*_@Au, and FeTaO_*x*_@Au-ANG NPs. The zero-field cooling (ZFC) and field cooling (FC) magnetization curves for these samples, as shown in Figure 4a, reveal that the direction of magnetization in the NPs is variable, with magnetization diminishing as the temperature rises. The blocking temperatures for FeTaO_*x*_, FeTaO_*x*_@Au, and FeTaO_*x*_@Au-ANG NPs were found to be 160, 150, and 124 K, respectively. When the temperature is higher than the blocking temperature, the ferromagnetic material loses its spontaneous magnetic properties, transforms from an ordered ferromagnetic phase to a disordered paramagnetic phase, and becomes superparamagnetic.^47^ Hence, all three materials are superparamagnetic. Figure 4b depicts the hysteresis curves (M–H curves) of FeTaO_*x*_, FeTaO_*x*_@Au, and FeTaO_*x*_@Au-ANG NPs. The saturation magnetization values of these materials are 8.99, 7.19, and 6.95 emu/gram, respectively. The slight decrease in saturation magnetization from FeTaO_*x*_ NPs (8.99 emu/g) to FeTaOx@Au-ANG NPs (6.95 emu/g) is attributable to the nonmagnetic Au coating and ANG modification, which reduce the overall magnetic content but do not compromise the superparamagnetic properties essential for MHT, collectively confirming the nanomaterial’s commendable therapeutic potential. Taken together, these results suggest that the conjugation of ANG to FeTaO_*x*_@Au NPs has little effect on their magnetic properties.

**Figure 4:**
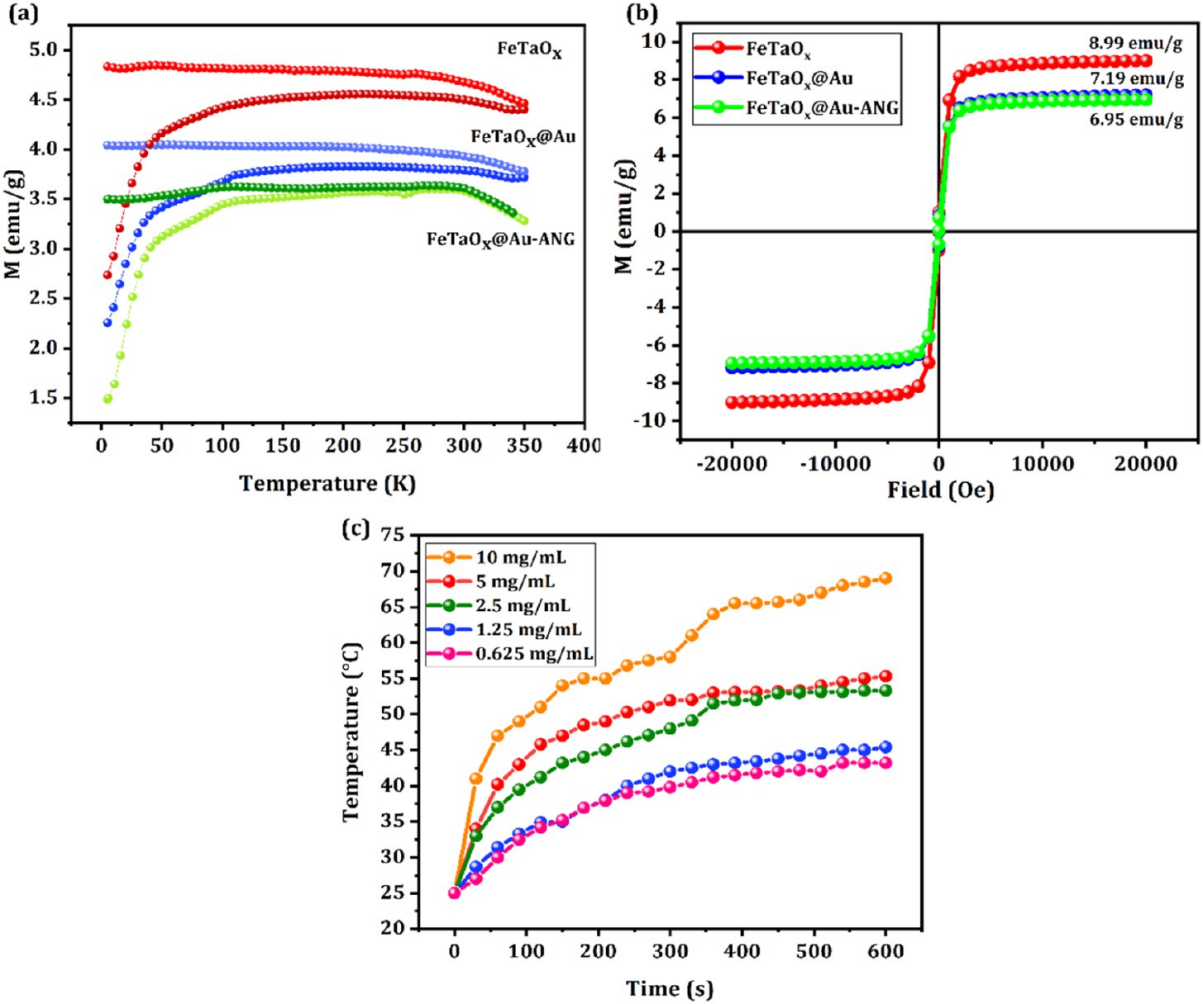
Magnetic Hyperthermic and SQUID analyses of FeTaO_*x*_, FeTaO_*x*_@Au, and FeTaO_*x*_@Au-ANG NPs. (a) ZFC/FC curves of FeTaO_*x*_, FeTaO_*x*_@Au, and FeTaO_*x*_@Au-ANG NPs. (b) Hysteresis curves (M–H curves) of FeTaO_*x*_, FeTaO_*x*_@Au, and FeTaO_*x*_@Au-ANG NPs. (c) Time-dependent temperature response of different concentrations of FeTaO_*x*_@Au-ANG NPs.

### 3.2 FeTaO_*x*_@Au-ANG NP’s Hyperthermia Property under Magnetic Stimulation

The hyperthermic responses of the synthesized FeTaO_*x*_@Au-ANG NPs were investigated using a high-frequency induction heating machine, Power Cube 3.2 kw (HF2). The FeTaO_*x*_@Au NPs generated heat under magnetic stimulation, and their respective IR images are shown in Figures 4c and S3. Previous studies have reported that cancer cells are sensitive to temperature variations.^52,53^ The carcinoma cells show signs of apoptosis and irregular behavior in their metabolism when they are exposed to a temperature range between 39 and 43 °C. In contrast, the normal cells remain stable at a temperature of around 46 °C. This temperature response of tumor cells makes them an indispensable tool for treating various cancers. To check the hyperthermic ability of the FeTaO_*x*_@Au NPs, the NPs’ were diluted to different concentrations (0.625, 1.25, 2.5, 5, and 10 mg/mL) and subjected to AMF. The findings showed that the heat generation was proportional to the concentration of the NPs. The temperature was maintained at approximately 35–42 °C for the 1.25 mg/mL concentration, highlighting that a small amount of NPs can generate sufficient heat for hyperthermia. Furthermore, the temperature increased with increasing concentration of NPs. This temperature response highlights the efficient role of FeTaO_*x*_@Au NPs in magnetic-field-stimulated hyperthermia for potential cancer treatment.

### 3.3 Multimodal Imaging Property

Iron-based materials possess good magnetic properties and can be used as contrast agents.^54^ Magnetic resonance spectroscopy was used to confirm the application of FeTaO_*x*_, FeTaO_*x*_@Au NPs, and FeTaO_*x*_@Au-ANG NPs as negative contrast agents. Iron possesses both *T*_1_ and *T*_2_ MRI properties. An inverse relationship between NPs concentration and signal intensity was observed. With increasing concentration of NPs, the color of the weighed MRI images (*T*_2_) changed from bright to dark, and the *T*_2_ signal intensity decreased with increasing Fe concentration. The findings illustrate the concentration-dependent MRI effect. A concentration of 0.0625 mg/mL of FeTaO_*x*_ and FeTaO_*x*_@Au NPs produced the highest intensity in MRI. FeTaO_*x*_@Au-ANG NPs displayed the lowest MRI intensity at 0.5 mg/mL. The MRI findings indicate that peptide conjugation to NPs can slightly decrease the MRI signal (Figure 5a). Figure 5b shows the concentration-dependent transverse relaxation times (*r*_2_) of the respective NPs. The relaxation time of FeTaO_*x*_,FeTaO_*x*_@Au and FeTaO_*x*_@Au-ANG NPs was 57.7, 59.4, and 47.7 mg^–1^ s^–1^, respectively. This decrease was attributed to the ANG peptide, which hindered the contact area between the NPs and the medium. The *T*_1_ MRI behavior of the prepared NPs was represented in Figure S4­(a); the color of the weighed images (*T*_1_) changed from dark to bright, highlighting the *T*_1_ MRI behavior of NPs; the *T*_1_ signal intensity increases along with an increase in NPs concentration. The *r*_1_ relaxivity of FeTaO_*x*_@Au-ANG NPs was determined by plotting the slope of the relaxation rate as proportional to the Fe concentration; the *r*_1_ values of FeTaO_*x*_@Au-ANG NPs were 56.189 mg^–1^ s^–1^, respectively, in Figure S4­(b). As a result, the prepared NPs hold dual-mode MRI contrast agents.

**Figure 5:**
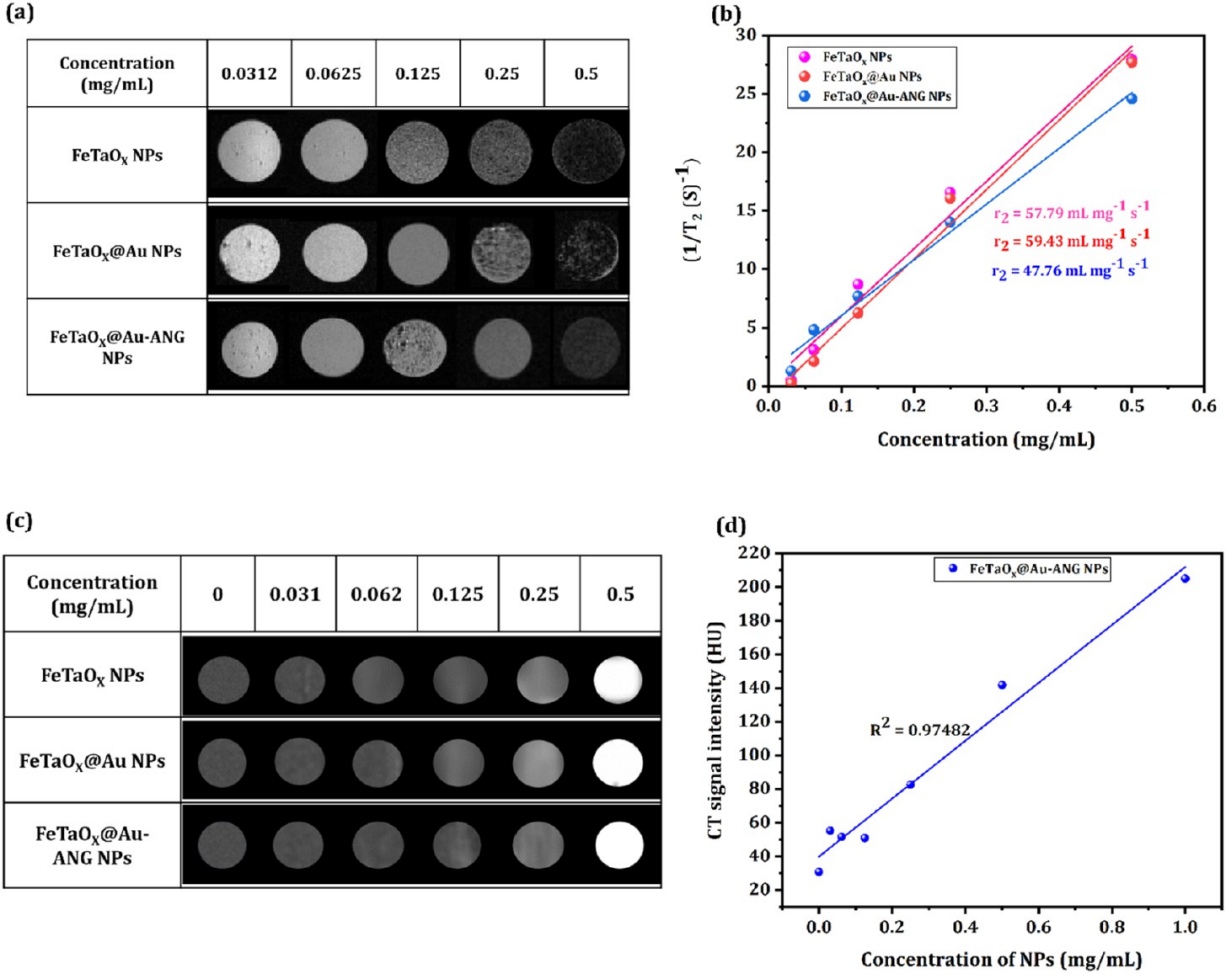
MRI and CT images of FeTaO_*x*_, FeTaO_*x*_@Au, and FeTaO_*x*_@Au-ANG NPs. (a) Various concentrations of NPs in agarose were used to examine the modulation of the image contrast (*T*_2‑_weighed MRI images). (b) Signal intensity for different groups. (c) CT images of the prepared NPs. (d) X-ray attenuation intensity (HU) values as a function of different concentrations of Au in NPs.

Multiple studies have reported that a dual-modality contrast agent is expected to have good clinical applications as a diagnostic tool.^55^ Both Fe and Au possess CT contrast properties. Additionally, Au has significant CT contrast ability compared to clinically used iodine-based CT contrast agents.^56–58^ NPs (FeTaO_*x*_@Au-ANG) enriched with Au were expected to provide good contrast to enhance X-ray CT.^59,60^ The X-ray attenuation CT potency of the FeTaO_*x*_@Au-ANG NPs with various concentrations of Au was examined. The results shown in Figure 5c indicate that the brightness of CT images obtained with aqueous solutions of FeTaO_*x*_@Au-ANG NPs increased with increasing NPs molar concentration. A linear increment of CT values of NPs with increasing NPs concentration in FeTaO_*x*_@Au-ANG NPs was observed (Figure 5d), confirming the potency of NPs as a good positive X-ray CT imaging contrast agent. These results also demonstrate the significant role of Au in NPs as a CT contrast agent, owing to its high X-ray absorption coefficient.

### 3.4 *In Vitro* Cytotoxicity Detection

To evaluate the *in vitro* cytotoxicity of FeTaO_*x*_@Au-ANG NPs, the CCK-8 assay was performed. The presence of Au in NPs is expected to result in good biocompatibility. L929 fibroblasts and C6 cells were used in this experiment. In Figure 6a, L929 cell viability results are shown. FeTaO_*x*_@Au-ANG NPs exhibited commendable cell growth inhibition than FeTaO_*x*_ and FeTaO_*x*_@Au NPs. FeTaO_*x*_@Au-ANG NPs used at 1 mg/mL displayed 82% biocompatibility. No significant decrease in cell viability was observed, indicating that ANG conjugation does not affect biocompatibility in L929 cells. However, the findings highlight the prospects of ANG use in the specific delivery of NPs to glioma (C6) brain tissue. C6 cells were cultured and incubated with different concentrations of NPs (31.25, 62.5, 125, 250, 500, and 1000 μg/mL). The concentration of FeTaO_*x*_@Au-ANG NPs was inversely proportional to cell viability (Figure 6b). Moreover, at 500 μg/mL, the cell viability decreased from 75% to 70% for FeTaO_*x*_@Au and FeTaO_*x*_@Au-ANG NPs. These results demonstrate that lower concentrations of NPs have fewer side effects on healthy stroma around the brain tissues. Compared to L929, the cytotoxicity of C6 cells due to NPs was high. At 1 mg/mL, the cell viability of FeTaO_*x*_@Au-ANG NPs toward L929 and C6 cells was estimated at 82% and 65%, mainly due to the higher uptake of NPs in the C6 cells by ANG conjugation. These results demonstrate that FeTaO_*x*_@Au-ANG NPs have good biocompatibility and excellent targeted cytotoxicity at tumor sites.

**Figure 6:**
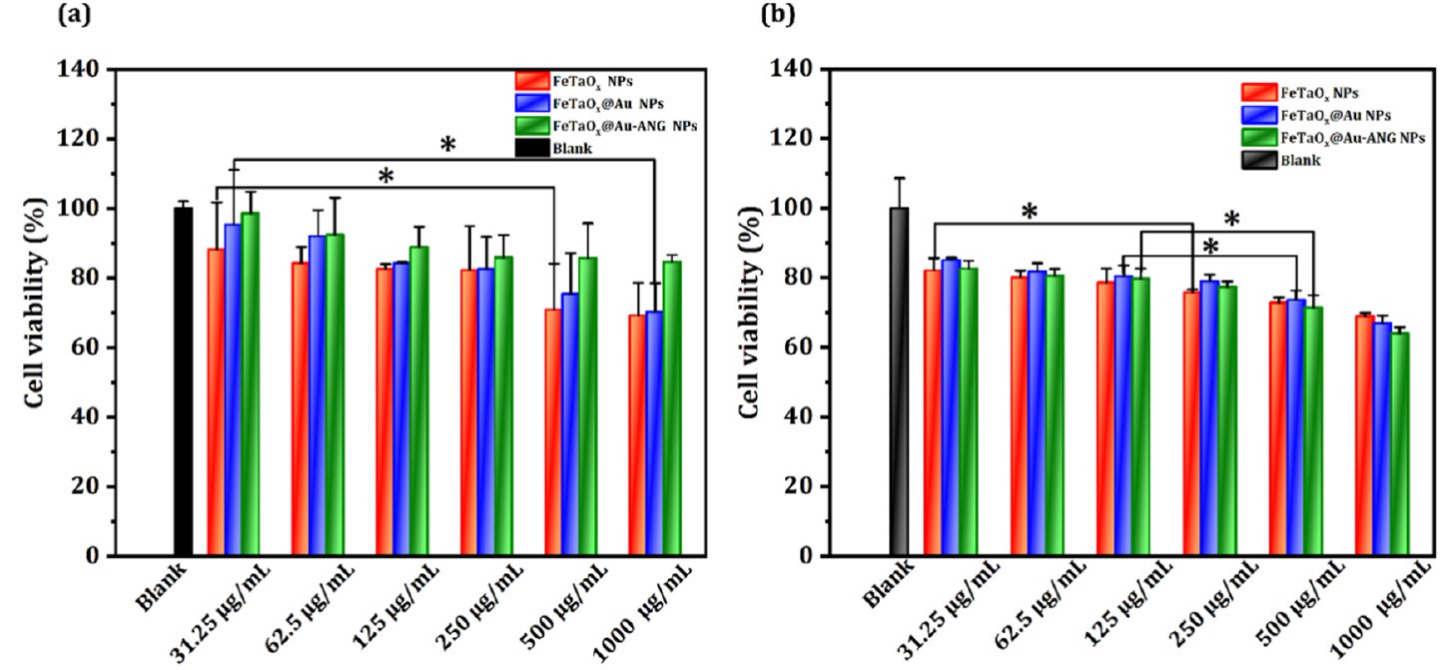
*In vitro* cytotoxicity analysis. Viability of L929 cells (a) and C6 cells (b) with different concentrations of FeTaO_*x*_@Au-ANG NPs. The cell viability studies show that the increased apoptosis rate of C6 cells than L929 cells because to hyperthermia treatment.

### 3.5 C6 Cell Uptake of FeTaO_*x*_@Au-ANG NPs

The modification of ANG on FeTaO_*x*_@Au NPs enhanced the cellular uptake of NPs toward C6 cells and induced apoptosis. To test this hypothesis, time-based optical images (Figure 7a–c) were obtained to validate the visualization of FeTaO_*x*_@Au and FeTaO_*x*_@Au-ANG NPs during endocytosis. C6 cells were cocultured with FeTaO_*x*_, FeTaO_*x*_@Au, and FeTaO_*x*_@Au-ANG NPs for 4 h and then subjected to optical imaging. The results revealed greater C6 cell endocytosis using FeTaO_*x*_@Au-ANG NPs compared to other groups, providing strong evidence that ANG conjugation makes the NPs target-specific to tumor cells. ICP-OES analysis was performed to evaluate the amount of Fe intake in L929 and C6 cells. L929 and C6 cells were cultured and incubated with FeTaO_*x*_, FeTaO_*x*_@Au, and FeTaO_*x*_@Au-ANG NPs for 1 and 4 h. C6 cells treated with FeTaO_*x*_@Au-ANG NPs at 4 h showed greater cellular uptake than L929 cells (Figure 7d), which was mainly due to the targeting of ANG-conjugated NPs toward LRP on C6 cells.

**Figure 7:**
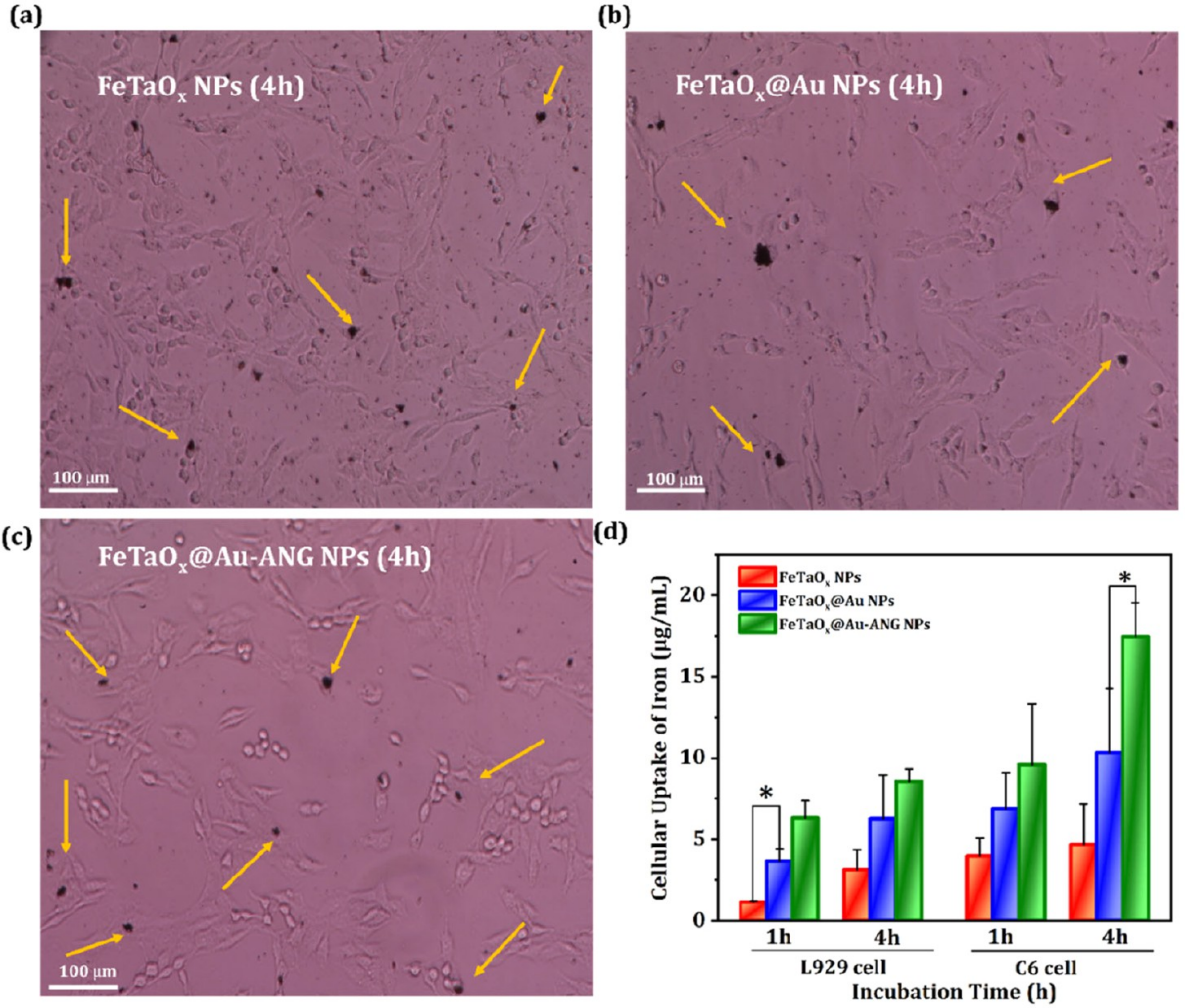
C6 cell uptake of NPs. (a–c) Optical microscopy images of the uptake of (a) FeTaO_*x*_, (b) FeTaO_*x*_@Au, and (c) FeTaO_*x*_@Au-ANG NPs during 4 h. (d) Graphical plot of ICP-OES analysis data of Fe concentration in the NPs incubated cells. The arrows in (a–c) indicate the localization of NPs.

### 3.6 AMF-Induced Hyperthermia Treatment of Glioma Cells

To elucidate the magetic hyperthermic performance of FeTaO_*x*_@Au-ANG NPs in ablating tumor cells, C6 cells were incubated with FeTaO_*x*_, FeTaO_*x*_@Au, and FeTaO_*x*_@Au-ANG NPs from 0 to 15 min (Figure 8). All experimental groups were subjected to AMF. Time-based magnetic stimulation was used to determine the optimal time required for magnetic field exposure to induce cell death. The cell survival rate gradually decreased with increasing magnetic field exposure time up to 10 min. FeTaO_*x*_@Au-ANG NPs exhibited excellent hyperthermia enhancement after 15 min of AMF treatment. After 15 min of treatment, C6 cell viability was decreased to 30%, 18%, and 9% with FeTaO_*x*_, FeTaO_*x*_@Au, and FeTaO_*x*_@Au-ANG NPs, respectively. The magnetic hyperthermia results indicated that a large number of tumor cells were necrotic because of the MHT treatment.

**Figure 8:**
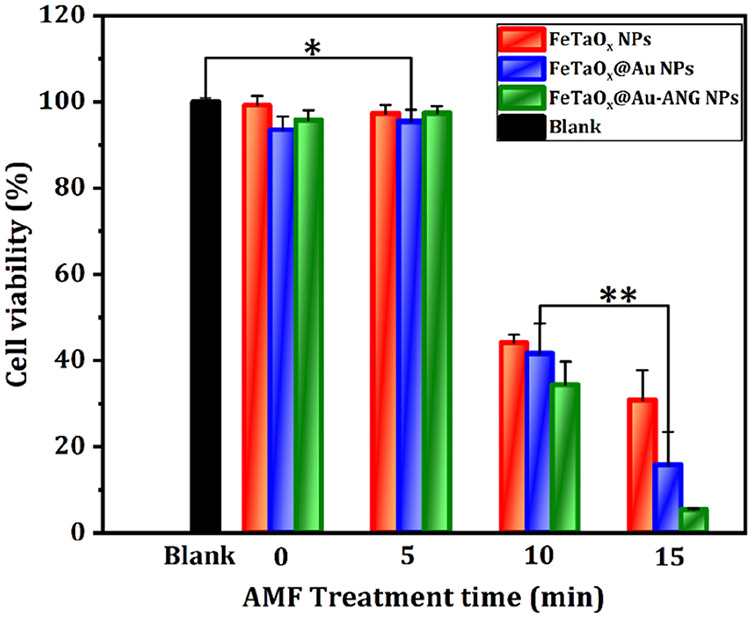
*In vitro* analysis of AMF-induced hyperthermia of FeTaO_*x*_, FeTaO_*x*_@Au, and FeTaO_*x*_@Au-ANG NPs in C6 cells.

### 3.7 Inductively Coupled Plasma Optical Emission Spectroscopy (ICP-OES) Analysis and Temperature Rise in Normal Brain Tissue

To further evaluate the accumulation and biodistribution of FeTaO_*x*_@Au-ANG NPs in rats, the concentrations of Fe and Ta in the major organs were measured by ICP-OES. To determine the uptake of NPs in each organ, ICP-OES was performed using an animal model without a tumor. For this experiment, the rats without tumors were taken and intravenously injected the 200 μL of NPs dispersed in 1 mL of PBS and were subjected to magnetic guidance. Later, the rat’s skull was opened, followed by the AMF treatment for 15 min, and the temperature rise in the normal brain was monitored by using an IR camera for 15 min (Figure S5). The results confirm that the normal brain experiences a temperature rise from 33.3 to 34.4 °C at the end of 15 min, highlighting the occurrence of localized hyperthermia effects in the single glioma cells or surrounding tissues, confirming that no severe temperature rise of the whole brain tissue takes place. The prepared NPs will not cause any damage to the normal cells. Figure S6­(a) conveys the ICP-OES studies, the presence of Fe atoms in different organs. Generally, every organ possesses Fe content naturally, to validate the uptake of NPs in the Brain, the Ta concentration in each organ was detected, and the results elucidated that compared to other organs (Heart, liver, spleen, lung, and kidney), the brain holds the highest Ta concentration (Figure S6­(b)).

### 3.8 Histological Analysis of *In Vivo* Hyperthermia Treatment of Glioma Tumors

Previous experiments in this study demonstrated that the synthesized NPs can act as good contrast MRI agents by modulating the *T*_2_ relaxation time (Figure 5a) and that brain tumor cells exhibit good specificity and ingestion of FeTaO_*x*_@Au-ANG NPs. Therefore, a rat model with predeveloped tumors was used to investigate the possibility of employing FeTaO_*x*_@Au-ANG NPs in brain tumor imaging. Figure 9a shows MRI images of tumor slices obtained at days 0, 7, and 14 for the control, FeTaO_*x*_, FeTaO_*x*_@Au, and FeTaO_*x*_@Au-ANG NPs groups. In the control rats, tumor volume rapidly increased with time. In contrast, the ANG-conjugated FeTaO_*x*_@Au NPs group displayed decreased tumor growth from day 0 to 14. The findings indicated that the Fe-based NPs conjugated with ANG can enter the glioma tumors and inhibit tumor growth by an excellent anticancer effect induced by hyperthermia. To elucidate the anticancer properties of NPs *in vivo*, SD rats were randomly divided into four groups. The control group of rats was intravenously injected with saline (0.2 mL). The rats in the FeTaO_*x*_, FeTaO_*x*_@Au, and FeTaO_*x*_@Au-ANG NPs groups were injected with 5 mg/mL, followed by hyperthermia (AMF). As shown in Figure 9b, the control, FeTaO_x,_ and FeTaO_*x*_@Au groups exhibited an enormous increase in tumor volume to 402, 271, and 185 mm_,_^3^ whereas the FeTaO_*x*_@Au-ANG NPs + AMF groups displayed decreased tumor volumes of 61.8 mm^3^. These results indicate that tumor growth is limited by hyperthermia-induced cell death triggered by Fe-based MNPs. Additionally, these findings demonstrated that even at low dosages, FeTaO_*x*_@Au-ANG NPs exhibit excellent *in vivo* therapeutic antitumor applications compared to the other groups. Additionally, we examined whether tumor volume reduction could translate into a higher survival rate (Figure 9c). Rats in the control group survived for 25 days, and rats administered FeTaO_*x*_ and FeTaO_*x*_@Au NPs survived for 30 and 32 days, respectively. Rats administered FeTaO_*x*_@Au-ANG NPs + AMF survived much longer (average of 50 days). The enhanced cellular uptake and apoptosis of ANG-conjugated FeTaO_*x*_@Au NPs can be attributed to the synergistic effect of ANG and FeTaO_*x*_@Au NPs in delivering cellular hyperthermia, resulting in tumor volume reduction and long-term survival. The *in vivo* anticancer potential of FeTaO_*x*_@Au-ANG NPs was evaluated because of the enhanced MHT process and higher ingestion of NPs by C6 cells. H&E staining was performed to examine the damage, pathological effects, and side effects that occurred in the tissue. H&E staining revealed that karyorrhectic tumor cells were covered by a large area of either a thick vascular wall with microvascular proliferation or coagulative necrosis (Figure 10a). Active proliferation of cells was evident in the control group; necrosis was observed in the FeTaO_*x*_ and FeTaO_*x*_@Au NPs groups, and necrosis was significantly increased in the FeTaO_*x*_@Au-ANG NPs group. The results confirm that active necrosis occurs because of elevated ingestion of NPs by cancer cells. Gliomas and anaplastic astrocytomas arise from astroglial cells. GFAP is the most common biomarker of astroglial cells. GFAP expression was lower in the control group. The highest number of GFAP-positive cells was observed in the ANG-conjugated NPs + AMF group. Additionally, dendrites were observed in these cells, and malignancy was attenuated in the FeTaO_*x*_@Au-ANG NPs + AMF group. Another frequently used cell proliferation indicator is the Ki67 labeling index, which positively correlates with a higher malignancy grade (the higher the Ki67 index, the faster the tumor growth). In the untreated (control) group, the Ki67 index value was 13.99%; the value gradually decreased as follows: 6.01% (FeTaO_*x*_), 3.96% (FeTaO_*x*_@Au), and 2.2% (FeTaO_*x*_@Au-ANG). Thus, we concluded that MHT can reduce the proliferative activity and malignancy of glioma cells. TUNEL staining confirmed the apoptosis of glioma cells by FeTaO_*x*_@Au-ANG NPs + AMF. Figure 10b shows H&E staining of vital organs. Rats bearing C6 glioma cells were sacrificed on day 14, and vital organs, including the heart, liver, lungs, spleen, and kidney, were removed and stained with H&E. The results in the control, FeTaO_*x*_, FeTaO_*x*_@Au, and FeTaO_*x*_@Au-ANG NPs indicated normal structure and morphology of the control cells. Cells in the FeTaO_*x*_, FeTaO_*x*_@Au, and FeTaO_*x*_@Au-ANG NPs groups also showed similar structures and morphologies to the control group, with no apparent tissue damage or abnormalities in the pathogenic tissue. Based on these findings, we concluded that FeTaO_*x*_@Au-ANG NPs possess no appreciable *in vivo* toxicity and have excellent antitumor effects.

**Figure 9:**
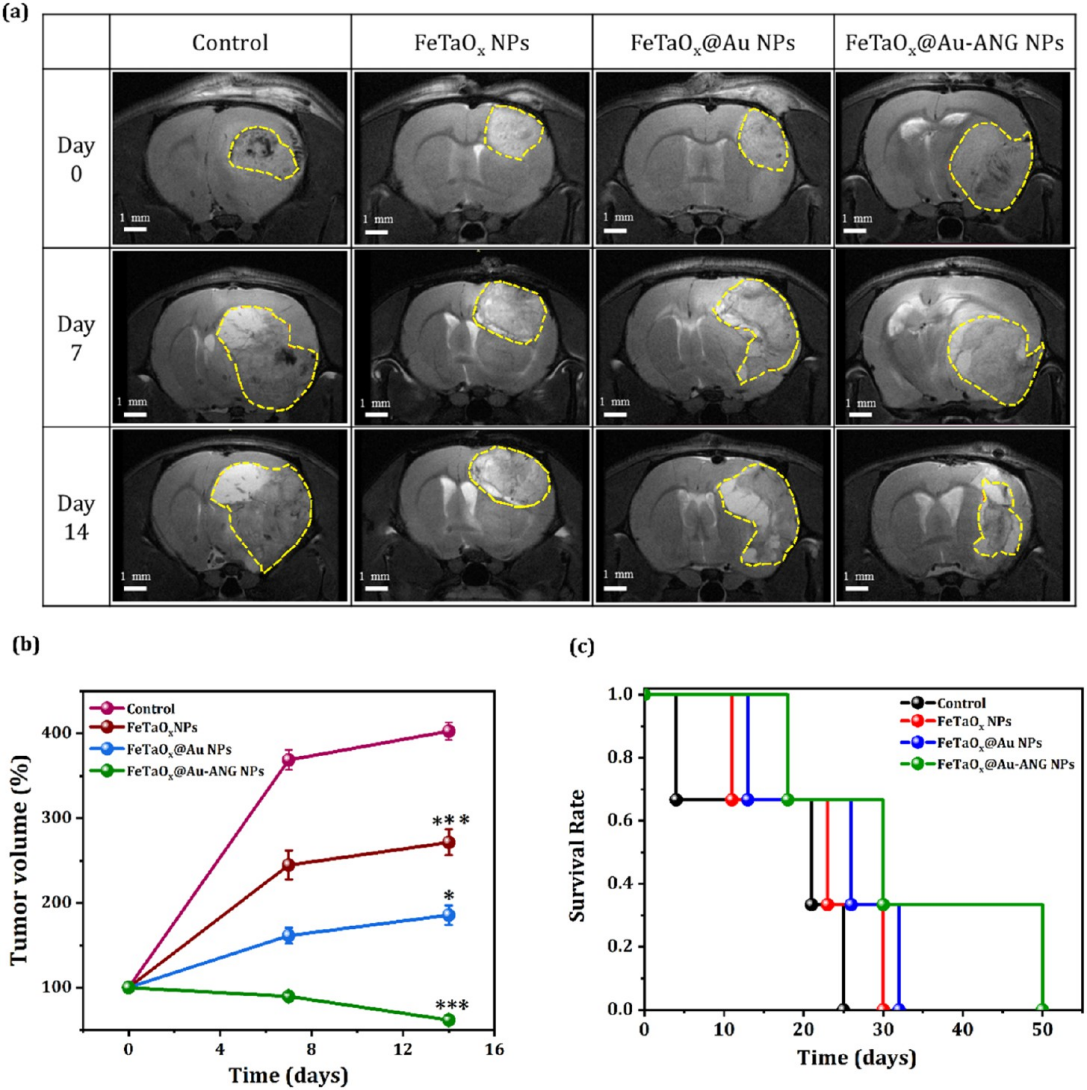
*In vivo* results. (a) MRI imaging of brain tumors of Sprague–Dawley rats 0, 7, and 14 days after FeTaO_*x*_@Au-ANG NPs injection with hyperthermia treatment. (b) Relative tumor volume curves of C6 tumor-bearing rats after the injection of saline (control) and other groups of NPs (*n* = 3). (c) Survival rate graph for the groups.

**Figure 10:**
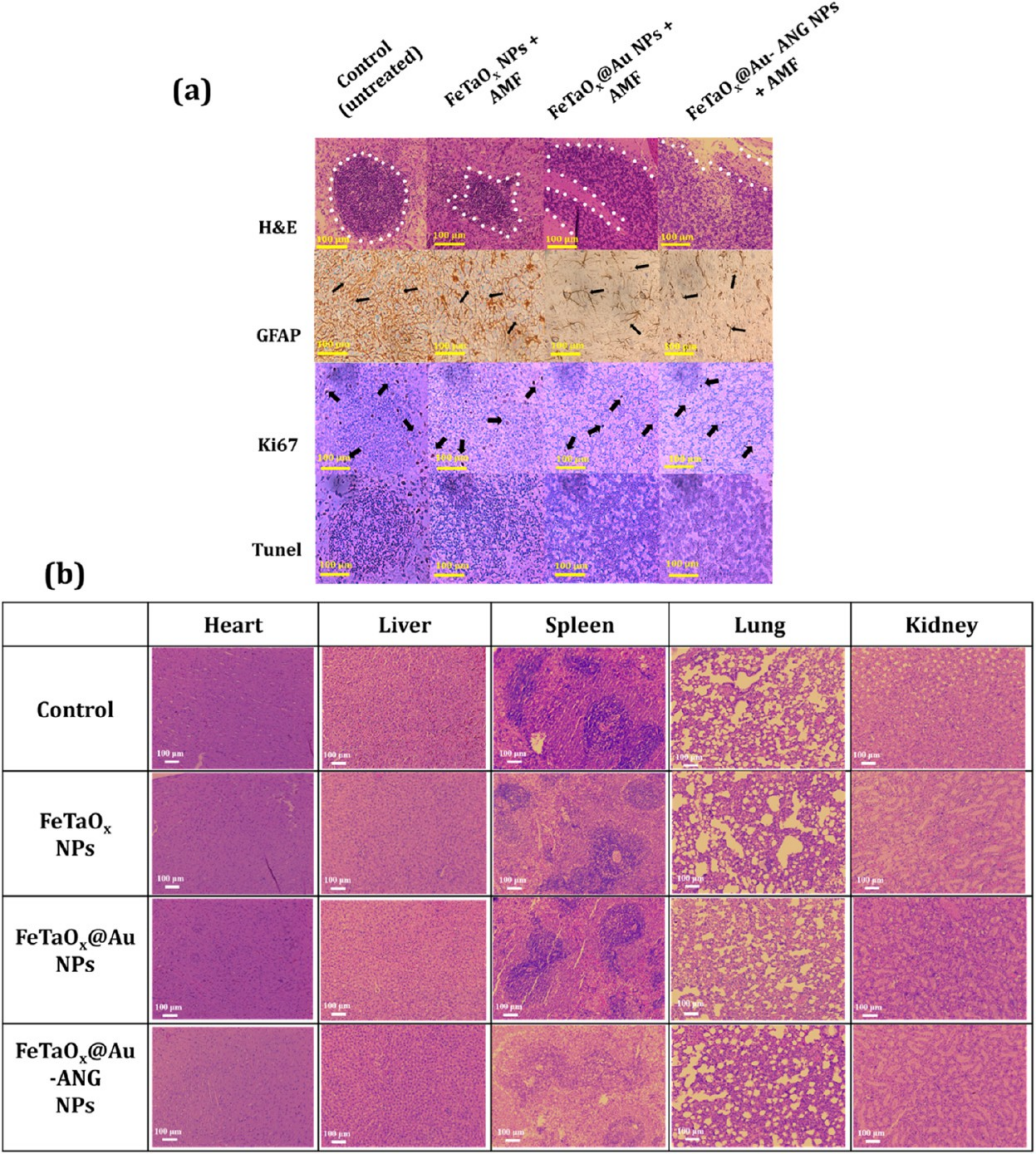
*In vivo* study data. (a) Immunohistochemistry analysis of tumor slices of the rat brain. The first row shows H&E staining. Active coagulative necrosis of tumor cells by ANG-conjugated NPs under AMF stimulation was evident. In the second row (GFAP staining), more GFAP-positive cells were observed in cells treated with FeTaO_*x*_@Au-ANG NPs and hyperthermia. In the third row (Ki67 staining), cells treated with FeTaO_*x*_@Au-ANG NPs decreased the Ki67 index after AMF stimulation, showing that hyperthermia limits glioma growth. The fourth row displays TUNEL-stained tumor tissue and (b) H&E staining images of vital organs, including the heart, liver, spleen, lung, and kidney, in the control, FeTaO_*x*_, FeTaO_*x*_@Au NPs, and FeTaO_*x*_@Au-ANG NPs groups. No damage or pathological changes were evident, suggesting no side effects. The black dots and arrows indicate the necrosis process (H&E), positive cell expression (GFAP), and Ki67 index level (Ki67), respectively.

## 4 Conclusions

In this study, we engineered FeTaO_*x*_ NPs and modified them using Au and ANG. FeTaO_*x*_@Au NPs conjugated with ANG allowed simultaneous tumor imaging and magnetic therapy to induce localized hyperthermia in the tumor cells. The affinity of ANG for the LPR receptor allowed ANG-conjugated NPs to easily cross the BBB. Heat generation was concentration-dependent owing to the superparamagnetic nature of the synthesized NPs. The inclusion of Fe and Au in NPs greatly improved the dual MRI and CT imaging properties. When tumor cells were exposed to FeTaO_*x*_@Au-ANG NPs coupled with AMF stimulation for 15 min, the *in vitro* data demonstrated approximately 95% cell apoptosis. Additional findings highlight that ANG-modified NPs are potential candidates that have outstanding performance in brain tumor therapy owing to their selective greater uptake by glioma cells compared to normal fibroblast cells. In addition, our *in vivo* studies revealed that when NPs were injected into rats with pre-existing tumors under AMF treatment, the prepared NPs showed the potential to generate hyperthermia. The platform designed in this study is extremely effective in halting tumor growth, and the survival rate of rats was increased up to 50 days.

The collective findings demonstrate that FeTaO_*x*_@Au-ANG NPs are well-suited for combined glioma imaging and therapy. Moreover, because of their simplicity, NPs can be conjugated with ANG to trigger MHT-induced cancer cell death in several cancer types. Many applications are likely to be derived from this research, including cancer biology, biomaterials, biomedical engineering, drug testing, drug development, and theranostics.

## Supplementary Material



## Data Availability

Data will be made available on request.

## Notes

EDX mapping and elemental composition of FeTaO_*x*_@Au-ANG NPs (Figure S1); stability study of NPs in PBS at different time intervals (Figure S2); IR thermal images of NPs under AMF exposure at varying concentrations and times (Figure S3); *T*_1_ MRI images of NPs (Figure S4); *in vivo* temperature elevation in normal brain under AMF with NPs (Figure S5); ICP-OES analysis of Fe and Ta biodistribution in major organs (Figure S6) (PDF)
