# Supplementary material for: Theranostic Angiopep-2-Conjugated FeTaO x @Au Core–Shell Magnetic Nanoparticles for Glioma Treatment and Dual Medical Imaging
Source: ACS Appl Bio Mater. 2026 Jan 6;9(2):1023–38. doi: 10.1021/acsabm.5c01925 (PMC12820972; doi:10.1021/acsabm.5c01925)
Supplement: Supplementary file 1 [file mt5c01925_si_001.pdf]

Supporting Information

Theranostic Angiopep-2-conjugated $FeTaO_x$@Au Core-Shell Magnetic Nanoparticles for Glioma Treatment and Dual Medical Imaging

Kayalvizhi Samuvel Muthiah [a,†], Senthilkumar Thirumurugan [a,†], Susaritha Ramanathan [a], Ming-Hsuan Yeh [a], Udesh Dhawan [b], Yu-Chien Lin [a,c], Ching-Po Lin [d], Wai-Ching Liu [e], Yuan-Yun Tseng [f,g,*], Ching-Li Tseng [h,i,j,k,*] , Ren-Jei Chung [a,l,*]

[a] Department of Chemical Engineering and Biotechnology, National Taipei University of Technology (Taipei Tech), Taipei 10608, Taiwan

[b] Centre for the Cellular Microenvironment, Division of Biomedical Engineering, James Watt School of Engineering, Mazumdar-Shaw Advanced Research Centre, University of Glasgow, Glasgow G116EW, UK

[c] School of Materials Science and Engineering, Nanyang Technological University, 50 Nanyang Avenue, Singapore 639798, Singapore

[d] Institute of Neuroscience, National National Yang Ming Chiao Tung University, Taipei 11221, Taiwan

[e] Department of Food and Health Sciences, Technological and Higher Education Institute of Hong Kong, Hong Kong 999077, Hong Kong

[f] Department of Neurosurgery, New Taipei Municipal TuCheng Hospital (Built and Operated by Chang Gung Medical Foundation), New Taipei City 236017, Taiwan

[g] College of Medicine, Chang Gung University, Taoyuan 33302, Taiwan

[h] Graduate Institute of Biomedical Materials and Tissue Engineering, College of Biomedical Engineering, Taipei Medical University, Taipei City 110, Taiwan

[i] International Ph. D. Program in Biomedical Engineering, College of Biomedical Engineering, Taipei Medical University, Taipei city 110, Taiwan

[j] Research Center of Biomedical Device, College of Biomedical Engineering, Taipei Medical University, Taipei city 110, Taiwan

[k] International Ph. D. Program in Cell Therapy and Regenerative Medicine, College of Medicine, Taipei Medical University, Taipei city 110, Taiwan

[l] High-value Biomaterials Research and Commercialization Center, National Taipei University of Technology (Taipei Tech), Taipei 10608, Taiwan

† These authors contributed equally to this paper.

* Corresponding Author: Dr. Ren-Jei Chung

Email: rjchung@mail.ntut.edu.tw; Tel: (886-2) 2771-2171 ext. 2547

Address: Department of Chemical Engineering and Biotechnology, National Taipei University of Technology (Taipei Tech), No. 1, Sec. 3, Zhongxiao E. Rd., Taipei 10608 Taiwan
Also corresponding to Prof. Yuan-Yun Tseng (britsey@gmail.com) and Prof. Ching-Li Tseng (chingli@tmu.edu.tw)

Index:

a. Figure S1. EDX analysis of the prepared NPs……………………………………………..S4

b. Figure S2. Stability study ………………………………………………………………....S4

c. Figure S3. IR images of AMF-treated NPs…………………………………..............S5

d. Figure S4. T_1-MRI images of NPs……………………………………………………...S5

e. Figure S5. *In vivo* temperature profile……………………………………………….....S6

f. Figure S6. ICP-OES studies of rat brain……………………………………………….S6

3. Results and Discussions

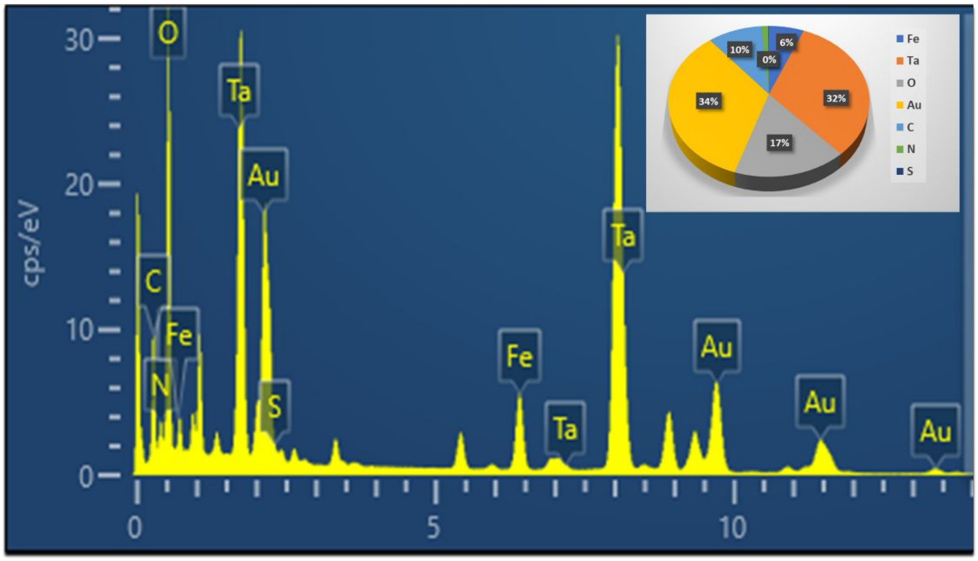

Figure S1.EDX mapping of $FeTaO_x$@Au-ANG NPs along with elemental composition and weight percentage.

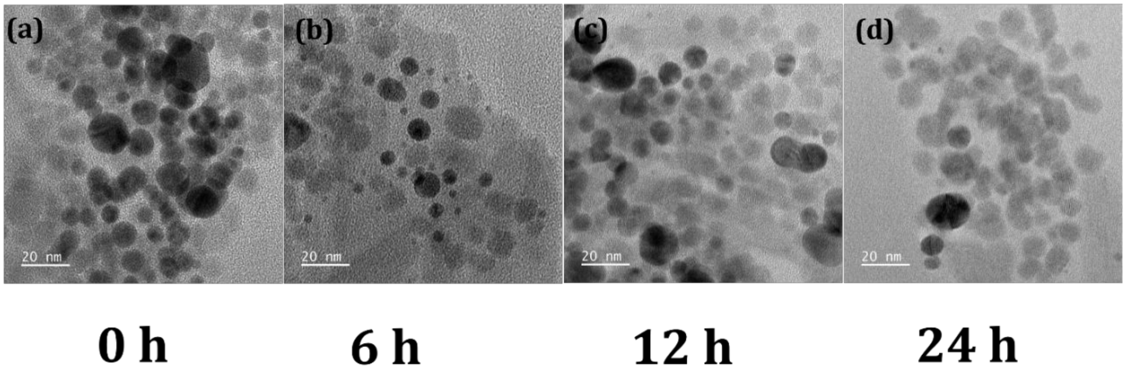

Figure S2: Stability study of NPs incubated in PBS solution for 0 h (a), 6 h (b), 12 h (c), and 24 h (d), respectively.

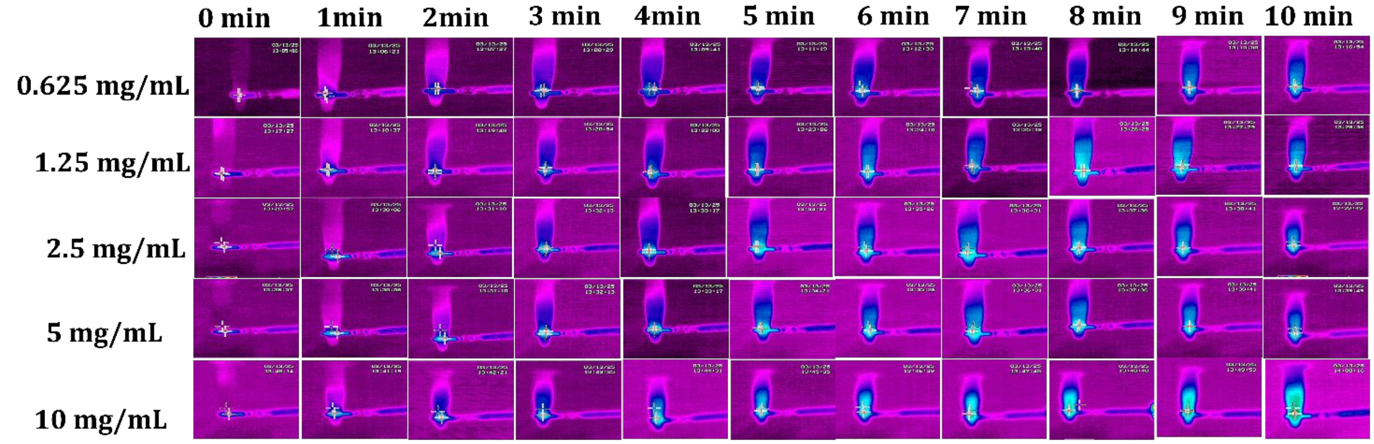

Figure S3: IR images of the different concentrations of the prepared NPs under AMF exposure for different time intervals (0-10 min) respectively.

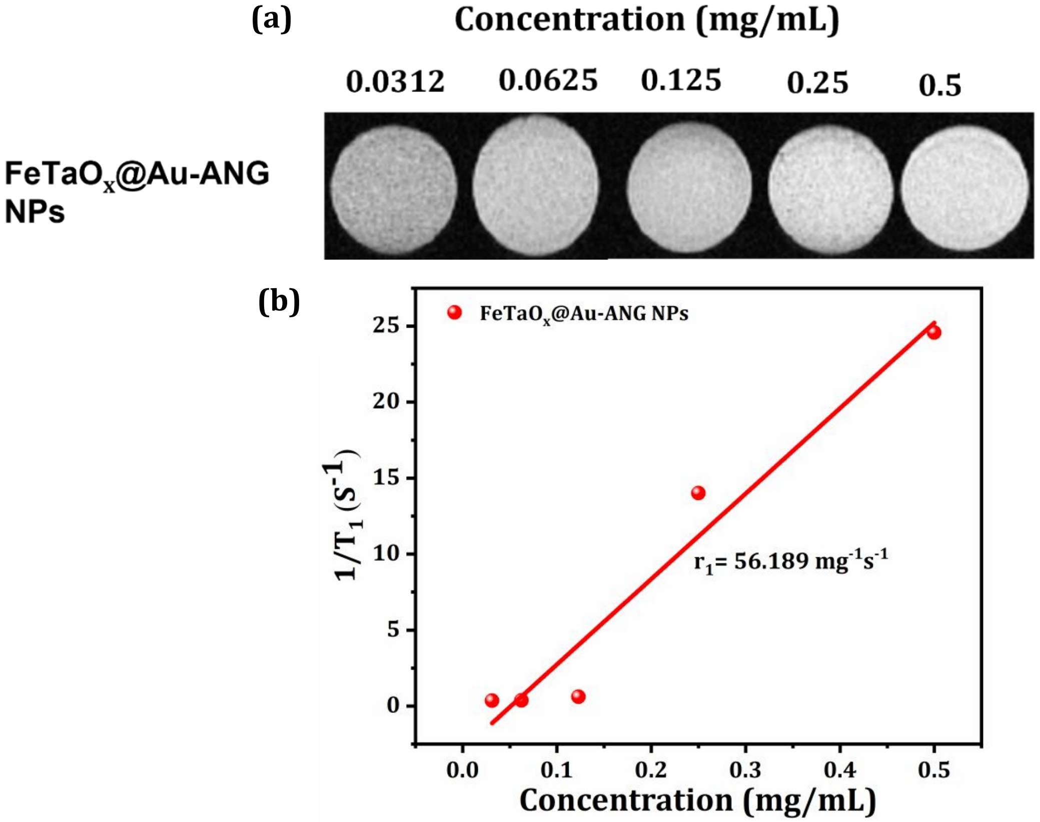

Figure S4: MRI behavior of NPs and signal intensity of NPs. (a) T_1-MRI images of the various concentrations of prepared FeTaO$_x$@Au-ANG NPs in agarose to investigate the image contrast modulation. (b) T_1- weighed MRI images concerning their signal intensity.

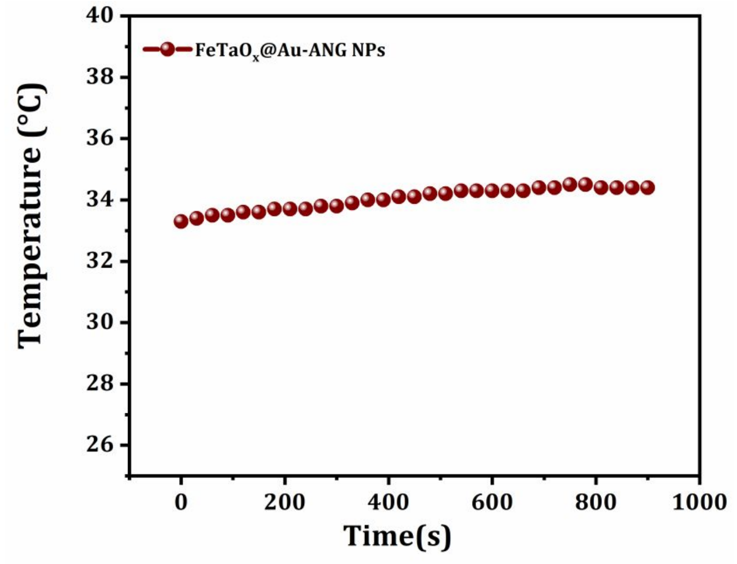

Figure S5: In *vivo* temperature elevation of the normal brain under the AMF conditions with NPs.

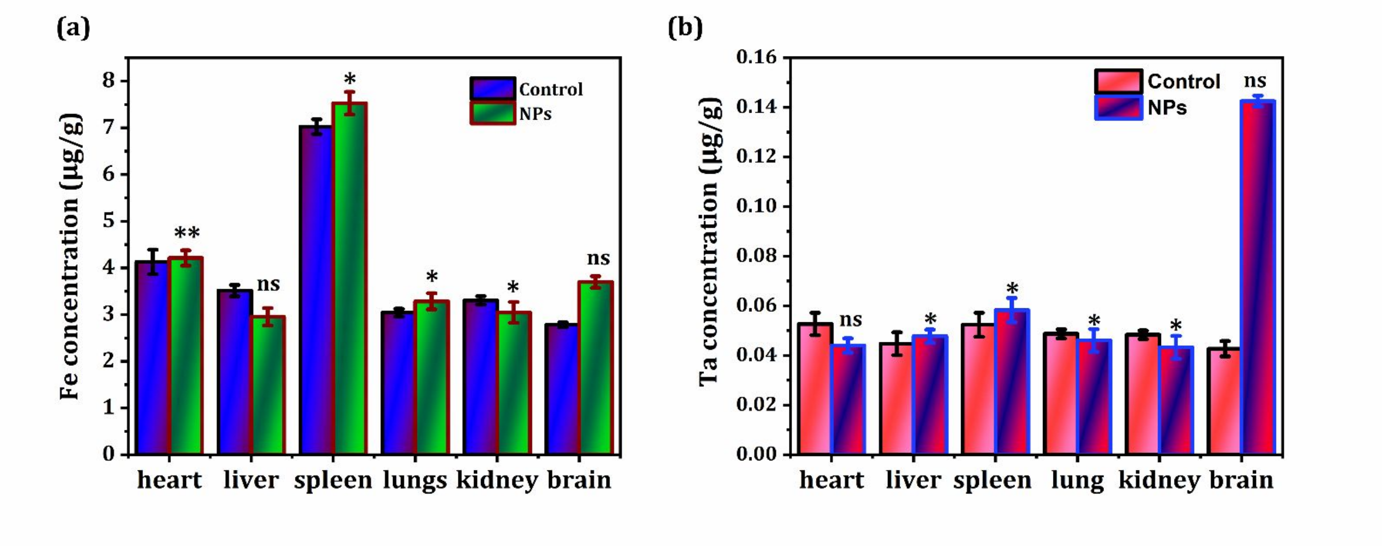

Figure S6: ICP-OES analysis showing the distribution of Fe and Ta in several major organs.
